# Largely Distinct Post‐Translational Modifications Differentiate Skeletal Muscle Wasting Caused by Cancer, Dexamethasone and Aging

**DOI:** 10.1002/jcsm.70220

**Published:** 2026-02-04

**Authors:** Anna Stephan, Flavia A. Graca, Suresh Poudel, Yingxue Fu, Yong‐Dong Wang, Myriam Labelle, Fabio Demontis

**Affiliations:** ^1^ Department of Developmental Neurobiology St. Jude Children's Research Hospital Memphis Tennessee USA; ^2^ Department of Immunology St. Jude Children's Research Hospital Memphis Tennessee USA; ^3^ Center for Proteomics and Metabolomics St. Jude Children's Research Hospital Memphis Tennessee USA; ^4^ Department of Cell and Molecular Biology St. Jude Children's Research Hospital Memphis Tennessee USA; ^5^ Division of Molecular Oncology, Department of Oncology St. Jude Children's Research Hospital Memphis Tennessee USA

**Keywords:** aging, atrophy, cancer cachexia, Leigh syndrome, Lrpprc, post‐translational modifications, sarcopenia, skeletal muscle weakness

## Abstract

**Background:**

Skeletal muscle wasting and weakness are prominent disease features. Originally considered to arise from common transcriptional changes, recent analyses demonstrated that different stimuli induce muscle wasting via largely distinct mRNA and protein changes.

**Methods:**

Here, we examined the post‐translational modifications (PTMs) associated with muscle wasting induced by cancer (*n* = 15 078), dexamethasone (*n =* 15 078) and aging (*n* = 8777) in mice by utilising the JUMPptm pipeline to recover modified peptides from TMT (tandem mass tag) mass spectrometry analyses.

**Results:**

We find that most PTMs that are significantly regulated are stimulus‐specific and that only a few are cross‐shared (*n* = 10; *p* < 0.05). These include P27 dihydroxylation of Lrpprc (leucine‐rich pentatricopeptide repeat containing), an RNA binding protein and transcriptional co‐activator mutated in Leigh syndrome, a mitochondrial disease. Contrary to the stimulus‐specificity of other atrophy‐associated PTMs, P27 dihydroxylation of Lrpprc declines (~20%; *p* < 0.05) with muscle wasting irrespective of the atrophic trigger. Electroporation of dihydroxylation‐resistant Lrpprc^P27A^ (which mimics the reduction in Lrpprc dihydroxylation that occurs with atrophy) reduces muscle force in young (~23%–39%; *p* < 0.01) and old (~26%–36%; *p* < 0.01) male mice compared to the contralateral electroporation of Lrpprc^WT^, indicating that a decline in Lrpprc P27 dihydroxylation contributes to muscle weakness in response to diverse catabolic stimuli. Comparison of Lrpprc^WT^ versus GFP electroporation indicates that there are mostly non‐significant effects (*p* > 0.05) on muscle force in young and old mice. Mechanistically, Lrpprc^P27A^ does not affect proteostasis and mitochondrial function compared to control Lrpprc^WT^ but impairs (> 60% decline; *p* < 0.05) the expression of genes necessary for muscle strength, including the apelin receptor *Aplnr* and *Col6a2/6* collagens. Moreover, Lrpprc^P27A^ reduces type 2b myofibre size (13% decline; *p* < 0.01) in old but not in young age.

**Conclusions:**

These analyses identify atrophy‐associated PTMs that provide refined biomarkers for fingerprinting the atrophic stimulus. Although most PTMs are stimulus‐specific, P27 dihydroxylation of Lrpprc declines during muscle wasting induced by cancer, dexamethasone and aging, suggesting that this is a general atrophy marker. Experimental up‐regulation of the atrophy‐mimicking variant Lrpprc^P27A^ reduces muscle force compared to wild‐type Lrpprc in young and old mice, suggesting that atrophy‐associated P27 dihydroxylation contributes to disease‐associated muscle weakness.

## 1 Introduction

Skeletal muscle wasting is a serious complication of many diseases and a side effect of common pharmacologic treatments but only limited therapeutic interventions are currently available. Skeletal muscle wasting associated with cancer is a component of cachexia [1], a systemic wasting syndrome that reduces the survival of cancer patients and their response to therapy [2, 3]. Notably, preventing muscle loss in tumour‐bearing mice improves their survival rates, even when cancer progression remains unaffected [2]. Cachexia‐associated wasting results from inflammatory cytokines produced by cancer cells and the surrounding tumour microenvironment, which promote tissue breakdown [1]. In addition to the marked muscle wasting that occurs with cancer and other disorders [4], muscle mass and function progressively decline during aging, a condition known as sarcopenia [5, 6] and in response to treatment with the glucocorticoid analogue dexamethasone [7], a commonly used anti‐inflammatory drug.

Muscle wasting entails a decline in protein synthesis and increased muscle proteolysis that leads to myofibre atrophy and muscle mass loss without any major changes in the number of myofibres [4]. The consequent decline in muscle mass is responsible for the corresponding induction of skeletal muscle weakness. In addition, conditions that typically do not lead to wasting such as chemotherapy and certain types of cancer (e.g., breast cancer) [8] can also induce muscle weakness.

A general mechanism that has been proposed to induce myofibre atrophy irrespective of the original stimulus entails the activation of signalling pathways that promote protein degradation and suppress protein synthesis [4]. On this basis, it was originally proposed that a common set of atrophy‐induced genes (‘atrogenes’) mediate the reduction in myofibre size that occurs during skeletal muscle wasting induced by different stimuli [9, 10, 11]. Although this model has identified key mediators of atrophy and has advanced research in this field, there are several indications of stimulus‐specific features of muscle wasting. Specifically, although dexamethasone and cancer cachexia induce a loss of muscle mass that correspondingly reduces force production, the decline in muscle strength that occurs with aging is typically ~3 times greater than simply explained by the reduction in muscle mass [5, 6], indicating that other mechanisms also contribute to sarcopenia. Indeed, recent studies have demonstrated that loss of protein quality control is a defining cause of sarcopenia compared to other types of muscle wasting that are disease‐associated [5]. Moreover, inflammatory signalling (via age‐associated circulating factors and cancer‐released inflammatory cytokines) and denervation contribute to muscle wasting during cachexia and sarcopenia but seemingly not in response to treatment with dexamethasone, an anti‐inflammatory drug that does not seem to cause overt denervation and that induces atrophy by activating glucocorticoid receptor signalling in myofibres [4]. Moreover, distinct myofibre types are differentially susceptible to atrophy induced by unrelated atrophic stimuli [4].

Besides these phenotypic differences, there is additional evidence that indicates that muscle wasting is not mediated by the same atrogenes irrespective of the inducing stimulus. Specifically, we have recently profiled the proteomic changes associated with muscle wasting at the endpoint of treatment with dexamethasone, cancer progression and aging and found that these are largely distinct, depend on the specific atrophic stimulus and do not necessarily correspond to the mRNAs that are modulated by these triggers of atrophy [12]. For example, the canonical atrogenes Fbxo32 and Trim63 were induced at the mRNA level by cancer and dexamethasone but not by aging, and the protein levels of these atrogenes were less modulated [12]. The disconnect between mRNA and protein changes indicates that proteomic markers of atrophy (‘atroproteins’) may be more reliable indicators of atrophy and of the specific inducing stimulus [12].

To further expand the proteomic repertoire of muscle‐wasting biomarkers, here we cross‐compared the post‐translational modifications (PTMs) that are associated with atrophy induced by cancer, dexamethasone and aging. To this purpose, we utilized the JUMPptm pipeline [13] to recover modified peptides from the whole TMT (tandem mass tag) mass spectrometry analyses.

Enrichment‐free proteomic analyses produce peptides that can bear PTMs that alter their mass compared to unmodified peptides. Analysis of the mass shifts of peptides can inform on the presence of multiple PTMs from enrichment‐free proteomic data. One of the approaches that can be used for these analyses is based on JUMPptm [13], a computational pipeline that utilizes JUMP and open search tools such as MSFragger to select PTMs and analyse them with Comet. Starting from TMT data, previous JUMPptm analyses identified PTMs that are differentially regulated during Alzheimer's disease [13], in response to perturbation of ubiquitin‐conjugating enzymes [14], and in *Drosophila* strains with exceptional longevity and preservation of skeletal muscle function during aging [15].

Here, we utilized JUMPptm to recover information on peptides that carry the eight PTM types that are typically most abundant and/or of general interest: acetylation, carboxylation, deamidation, dihydroxylation, methylation, oxidation, phosphorylation and ubiquitination. We find that most PTMs that are induced during skeletal muscle wasting are stimulus‐specific and only minimally cross‐shared. One of the few commonly regulated PTMs is the decline in proline 27 dihydroxylation of Lrpprc (leucine‐rich pentatricopeptide repeat containing), also known as Lrp130 (leucine‐rich protein 130 kDa). The *Lrpprc* gene is mutated in Leigh syndrome [16, 17, 18], a congenital mitochondrial disease characterized by cytochrome oxidase deficiency [19, 20, 21]. On this basis, we tested the impact on muscle function of wild‐type Lrpprc compared to a dihydroxylation‐resistant P27A mutant, which mimics the reduction in P27 dihydroxylation that occurs during muscle wasting induced by multiple stimuli. We find that Lrpprc^P27A^ reduces muscle strength (without affecting muscle mass) compared to Lrpprc^WT^ in young and old mice. Altogether, these findings indicate that a reduction in Lrpprc P27 dihydroxylation is a widespread (and potentially general) marker of muscle atrophy that is consistently modulated irrespective of the atrophic stimulus. Our finding that the hydroxylation‐resistant variant Lrpprc^P27A^ reduces muscle force suggests that the atrophy‐associated reduction in Lrpprc P27 dihydroxylation contributes to the decline in muscle strength that occurs during muscle wasting.

In summary, our large‐scale resource provides a global view of the PTMs that are associated with stimulus‐specific muscle wasting. We propose that such PTMs may provide additional biomarkers for muscle wasting, and that some of these wasting‐associated PTMs may also contribute to myofibre atrophy and/or to the decline in muscle strength induced by cancer, dexamethasone and aging.

## 2 Methods

### 2.1 JUMPptm Identification and Statistical Analysis of Differentially Expressed Peptides With PTMs

The JUMPptm search [13, 15, 22] was conducted on the TMT proteome datasets we previously described [12] and that are available at the ProteomeXchange Consortium via the PRIDE partner repository under accession numbers PXD027464 and PXD027490. High‐quality, unmatched spectra with de novo tags obtained post‐JUMP search for each TMT set were utilized as input for JUMPptm, as previously described [13]. This input was searched against eight major PTMs (acetylation, carboxylation, deamidation, dihydroxylation, methylation, oxidation, phosphorylation and ubiquitination) using a multi‐stage database search strategy. The resulting peptides with PTMs were quantified using the TMT‐based quantification function of the JUMP software suite. Differentially expressed peptides with PTMs were identified using the limma package in R Studio. The *p* values were computed using a moderated *t* test, and the false discovery rate (FDR) values were determined using the Benjamini–Hochberg procedure (see Datasets S1 and S2).

### 2.2 PTM Consensus Sequences

**I**ceLogo was utilized to examine the amino acid frequencies surrounding the sites of PTMs. Input data consisted of 15‐residue sequences centred on the modified residue, which were compared to the reference *mouse* amino acid composition dataset from Swiss‐Prot. A percentage‐based scoring system was applied, and significant changes (*p* < 0.05) were visualized (see Dataset S3).

### 2.3 Electroporation of DNA Plasmids Into the Tibialis Anterior Muscle

C57BL/6 J male mice (The Jackson Laboratory, JAX#000664) were housed in the Animal Resource Center at St. Jude Children's Research Hospital, fed a standard chow diet and handled following protocols approved by the St. Jude Children's Research Hospital Institutional Animal Care and Use Committee (IACUC).

Tibialis anterior (TA) muscles of 4‐month‐old (‘young’) and 27‐month‐old (‘old’) male C57BL/6 J mice were electroporated with DNA plasmids following the procedures previously reported [23]. One TA was electroporated with the pCMV6‐Lrpprc^WT^‐3xFlag plasmid, which encodes for Flag‐tagged wild‐type mouse Lrpprc, whereas the contralateral TA was electroporated with the pCMV6‐Lrpprc^P27A^‐3xFlag plasmid, which encodes for a Flag‐tagged mouse Lrpprc variant with a P27A mutation. Proline to alanine modification was previously shown to render proline refractory to hydroxylation [24]. Other mice were electroporated with a control GFP plasmid. For the electroporation of these plasmids, the mice were first anaesthetised with isoflurane, the hair was removed from the hind legs, and the TA was then injected with 30 μL of 0.4 U/μL hyaluronidase (Sigma #4272) using a 29G1/2 insulin syringe. After recovering for 2 h, the mice were anaesthetised again and 40 μL of DNA were injected in PBS (at 1 μg/μL) into the TA muscle followed by electroporation by using an Electro Square Porator (BTX Harvard Apparatus #ECM830) and electrodes (BTX Harvard Apparatus Genetrodes, Straight, 10 mm Gold Tip #45‐0114) placed parallel to the tibia orientation. Specifically, four pulses at 80 V/cm with 20‐ms length at 1 Hz were delivered, followed by another four pulses after the orientation of the electrodes was switched perpendicular to the tibia. After 14 days from electroporation, the mice were sacrificed, and the TA muscles were dissected and snap‐frozen for subsequent analyses.

### 2.4 qRT‐PCR

qRT‐PCR was performed as previously described [23]. Total RNA was extracted with the TRIzol reagent (Ambion #15596018) followed by reverse transcription with the iScript cDNA synthesis kit (Bio‐Rad). qRT‐PCR was performed with SYBR Green and a CFX96 apparatus (Bio‐Rad). The comparative C_T_ method was used for the relative quantitation of mRNA levels normalized based on the *Hprt* levels. The following qRT‐PCR oligos were used:

*Lrpprc*: 5′‐CTCCCGCTGTCCCTGCGCCTCC‐3′ and 5′‐CAGTCTCATCAGAGCCCAGTCAAATTGAC‐3′.

*Hprt*: 5′‐GATTAGCGATGATGAACCAGGTT‐3′ and 5′‐TCCAAATCCTCGGCATAATGAT‐3′.

### 2.5 RNA Sequencing

RNA sequencing samples were prepared using TRIzol (Ambion #15596018) from the TA skeletal muscle, followed by RNA extraction via isopropanol precipitation from the aqueous phase. Libraries for RNA sequencing were generated from 1 μg of total RNA using the Illumina TruSeq RNA Sample Prep v2 Kit, according to the manufacturer's guidelines. Sequencing was performed on the Illumina NovaSeq 6000 platform, producing 100‐bp paired‐end reads. These reads were processed by trimming and filtering based on quality (Phred‐like Q20 or higher) and length (≥ 50 bp) and subsequently aligned to the mouse reference genome (GRCm39/mm39) using CLC Genomics Workbench v20.0.4 (Qiagen). For gene expression analysis, TPM (transcripts per million) values were derived using the CLC RNA‐Seq Analysis tool. Differential gene expression was assessed using a non‐parametric ANOVA, applying Kruskal–Wallis and Dunn's tests on log‐transformed TPM values, implemented in Partek Genomics Suite v7.0 (Partek Inc.). Gene Ontology (GO) term analysis was done using webgestalt (https://www.webgestalt.org/) and DAVID (https://davidbioinformatics.nih.gov/tools.jsp).

The RNA‐seq data of gene expression changes induced by Lrpprc^P27A^ vs. Lrpprc^WT^ (see Dataset S4) were deposited to the GEO and are accessible with the identifier GSE296017. The RNA‐seq data from mouse muscles with different types of atrophy (aging, cancer and dexamethasone) were previously published [12] and are accessible at the GEO with the identifier GSE159952.

### 2.6 Skeletal Muscle Force Measurements

The measurement of the twitch and tetanic force of the TA muscle was done as previously described [23, 25]. Mice were deeply anaesthetised via isoflurane and monitored throughout the experiment. The distal tendon of the TA was carefully dissected and individually tied with braided surgical silk (12, 5/0, 19 mm blade, ½ reverse cutting). The sciatic nerve was exposed, and all branches were cut except for the common peroneal nerve. The foot was secured to a platform, and the knee was immobilized using a stainless‐steel pin. The body temperature was monitored and maintained at 37°C. The suture from the tendon was individually attached to the lever arm of a 305B dual‐mode servomotor transducer (Aurora Scientific, Ontario, Canada). Muscle contractions were then elicited by stimulating the distal part of the sciatic nerve via bipolar electrodes, using supramaximal square‐wave pulses of 0.2 ms (701A stimulator; Aurora Scientific). Data acquisition and control of the servomotor were conducted using a Lab‐View‐based DMC program (version 5.202; Aurora Scientific). Optimal muscle length (Lo) was determined by incrementally stretching the muscle until the maximum isometric twitch force was achieved. The fatigue resistance protocol consisted of 60 tetanic contractions (60‐Hz stimulation/500‐ms duration) every 3 s for a total of 3 min.

### 2.7 Immunostaining and Confocal Microscopy of Skeletal Muscles

The analysis of myofibre size, type and number was done according to the previously reported procedures [23, 25, 26, 27]. Slides with unfixed sections of TA muscles were incubated for 1 h in a blocking buffer consisting of PBS with 2% BSA and 0.1% Triton X‐100, followed by overnight incubation at 4°C with primary antibodies diluted 1:150. The primary antibodies used consisted of mouse IgG1 anti‐myosin heavy chain Type 2a (DSHB, #SC‐71), mouse IgM anti‐myosin heavy chain Type 2b (DSHB, #BF‐F3) and rat anti‐laminin α2 (4H8‐2; Santa Cruz, #sc‐59 854). After washing, sections were incubated with secondary antibodies (1:200): anti‐mouse IgG1 Alexa Fluor 488 (Life Technologies, #A21121), anti‐mouse IgM Alexa Fluor 555 (Life Technologies, #A21426) and anti‐rat IgG Alexa Fluor 647 (Life Technologies, #A21247). Imaging was done using a Nikon confocal microscope with a 10x objective, and the images were stitched to obtain an overview of the muscle cross‐section. Myofibre size and type were automatically analysed using the Nikon Elements software. Myofibre boundaries were delineated based on the inverse threshold of laminin α2 immunostaining, and fibre types were identified based on the myosin heavy chain staining: Type 2a (green), Type 2b (red) and presumed Type 2x (unstained, appearing black). Myofibre size was estimated automatically with the Feret's minimal diameter, a geometrical measure suitable for irregularly shaped or sectioned myofibres. To quantify the total myofibre number, all myofibres in the muscle cross‐sections were automatically counted based on the borders defined by laminin α2 staining.

### 2.8 Seahorse Assays From Cultured Myofibres

After 2 weeks from electroporation, the TA muscle was dissected and placed in Tyrode's solution (5.4 mM KCl, 1 mM MgCl_2_, 140 mM NaCl, 0.33 mM NaH_2_PO_4_, 2 mM CaCl_2_, 10 mM d‐glucose and 10 mN HEPES [pH 7.4]). Subsequently, the TA muscles were placed in 12‐well plates containing a collagenase solution (4 mg/mL, #10103586001) for 1 h at 37°C. Following the dissociation, the muscle was placed in a new 12‐well plate and washed twice with DMEM (Gibco, #21063‐029) containing antimycotics and antibiotics. To obtain single myofibres, the same amount of TA muscle for each condition was transferred to a new 12‐well plate with warm DMEM medium containing 10% FBS. The fibres were dissociated from the TA muscle using a wide‐bore tip (ART 1000G Aerosol Resistant Tip, #2079G) and by gently flushing the medium. After dissociation, the plate was kept overnight in an incubator at 37°C. In preparation for seeding the dissociated myofibres, the Agilent Seahorse X96 cell culture microplate (Agilent Technologies, #103792‐100) was coated with 10 μL of growth factor reduced Matrigel matrix (Corning, #356230) diluted 1:2 in DMEM. The plate was then placed in an incubator at 37°C for 1 h. Following a thorough dispersion of single myofibres, 25 μL aliquots were taken randomly and deposited into each well, where they were attached by sedimentation to allow the myofibres to cover ~60% of the well bottom. The plate was then placed in an incubator overnight. On the day of the assay, the medium was exchanged with Seahorse XF DMEM medium, pH 7.4 (Agilent Technologies, #103792‐100) supplemented with 4.5 mM d‐glucose (Sigma‐Aldrich, #G8270), 2 mM sodium pyruvate (Gibco, #11360‐070) and 2 mM l‐glutamine (Gibco, #25030‐081), and then the plate was placed in an incubator at 37°C for 1 h. Meanwhile, oligomycin A (Oligo, #J61898.MA), carbonyl cyanide‐*p*‐trifluoromethoxyphenylhydrazone (FCCP, #C2920‐10 mg) and antimycin (AA, #HY‐B1756) plus rotenone (Rot, #J63522.MA) were prepared with assay medium and loaded into ports A, B and C of the XFe 96 sensor cartridge (20, 22 and 25 μL, respectively). The final working concentrations of oligomycin A, FCCP and antimycin‐rotenone were 2, 2 and 0.5 μM, respectively. The oxygen consumption rate (OCR) was continuously recorded for 12 cycles, with each cycle consisting of 3 min of mix, 30 s of wait and 3 min of measurement. The first three cycles were utilized to measure the basal respiration. Subsequently, three cycles were each run following the injection of Oligo, FCCP and Rot‐AA. These procedures are a modification of previously published protocols established for isolated myofibres [28, 29], and we observed responses to drug treatments similar to those previously reported for this system. Normalisation is ensured by the preparation of myofibres from equal amounts of TA muscles and by plating equivalent numbers of myofibres across conditions. Because Matrigel is used for plating the myofibres, a normalisation based on protein content is not possible in this assay.

### 2.9 Complex I and Complex IV Activity Assays

Mitochondrial Complex I and Complex IV activities were, respectively, probed in muscle homogenates with the CheKine micro mitochondrial Complex I activity assay kit (Abbkine, #KTB1850) and the CheKine micro mitochondrial Complex IV activity assay kit (Abbkine, #KTB1880) and normalized by protein content with the BCA protein assay kit (Thermo Fisher #A53227).

### 2.10 Western Blotting

TA muscles were homogenized in 200 μL NP40 cell lysis buffer (Invitrogen, #FNN0021), and the protein extracts were quantified by using the Bradford assay (Bio‐Rad protein assay dye reagent concentrate, #5000006). Protein samples were prepared for SDS‐PAGE by adding SDS‐Blue loading buffer (Cell Signalling, #7722) and 0.1 M DTT (Cell Signalling, #1425 S) and by heating the samples at 95°C for 5 min. Samples were then run on 4%–20% gradient SDS‐PAGE gels (Bio‐Rad, #4561096) alongside a molecular weight ladder (Bio‐Rad, #1610374) and transferred to PVDF membranes (Millipore, #IPVH00010), which were blocked with either 5% milk powder or 5% BSA for 1 h. Subsequently, the membranes were sequentially incubated overnight at 4°C with primary antibodies (at 1:1000) for PGC‐1α (Invitrogen, #PA5‐72948) and α‐tubulin (Cell Signalling, #2125). For the analysis of proteostasis markers, the following primary antibodies were used: anti‐ubiquitin (P4D1, Santa Cruz Biotechnology #sc‐8017), anti‐phospho‐Atg16 (Abcam #ab195242) and anti‐LC3 (rabbit polyclonal anti‐LC3 Sigma #L7543). In a different experiment, the samples were prepared as described above but not heated, followed by SDS‐PAGE and Western blot with an antibody cocktail that detects the assembly status of the electron transport chain (1:1000; rodent OXPHOS Cocktail, Abcam ab#110413). This assay is based on the capacity of each antibody in the cocktail to recognize a labile subunit (i.e., NDUFB8, SDHB, UQCRC2, MTCO1 and ATP5A) that is stable only when part of an OXPHOS complex (i.e., respectively of Complex I, II, III, IV and V). The full scans of Western blots are provided in the Source Data file (Dataset S5).

### 2.11 Statistical Analyses

Data organisation, scientific graphing and statistical analyses were performed with Microsoft Excel (version 14.7.3), GraphPad Prism (version 10) and Srplot (https://www.bioinformatics.com.cn/srplot). The two‐tailed Student's *t* test or Welch's *t* test (as indicated in the figure legend) was used to compare the means of two independent groups with each other. Two‐way ANOVA with Tukey's post hoc testing was used for multiple comparisons when two independent variables were present. The n for each experiment can be found in the figure legends and represents independently generated samples sourced from distinct mice. Bar graphs display the mean ± SD or ± SEM, as indicated in the figure legend. A significant result was defined as *p* < 0.05. Throughout the figures, asterisks indicate the significance of *P* values: *p < 0.05, **p < 0.01 and ***p < 0.001.

## 3 Results

### 3.1 Identification of Post‐Translational Modifications That Occur During Skeletal Muscle Wasting Induced by Cancer, Dexamethasone and Aging in Mice

We previously profiled the proteomic changes that occur in the TA skeletal muscle of male C57BL/6 J mice at the endpoint of treatment with the glucocorticoid analogue dexamethasone, cancer cachexia (due to the subcutaneous injection of Lewis lung carcinoma [LLC] cells) and aging (24 vs. 6 months) [12]. These deep‐coverage TMT mass spectrometry analyses provided key insight into the protein biomarkers that are associated with and that may contribute to skeletal muscle wasting by distinct stimuli [12].

**FIGURE 1:**
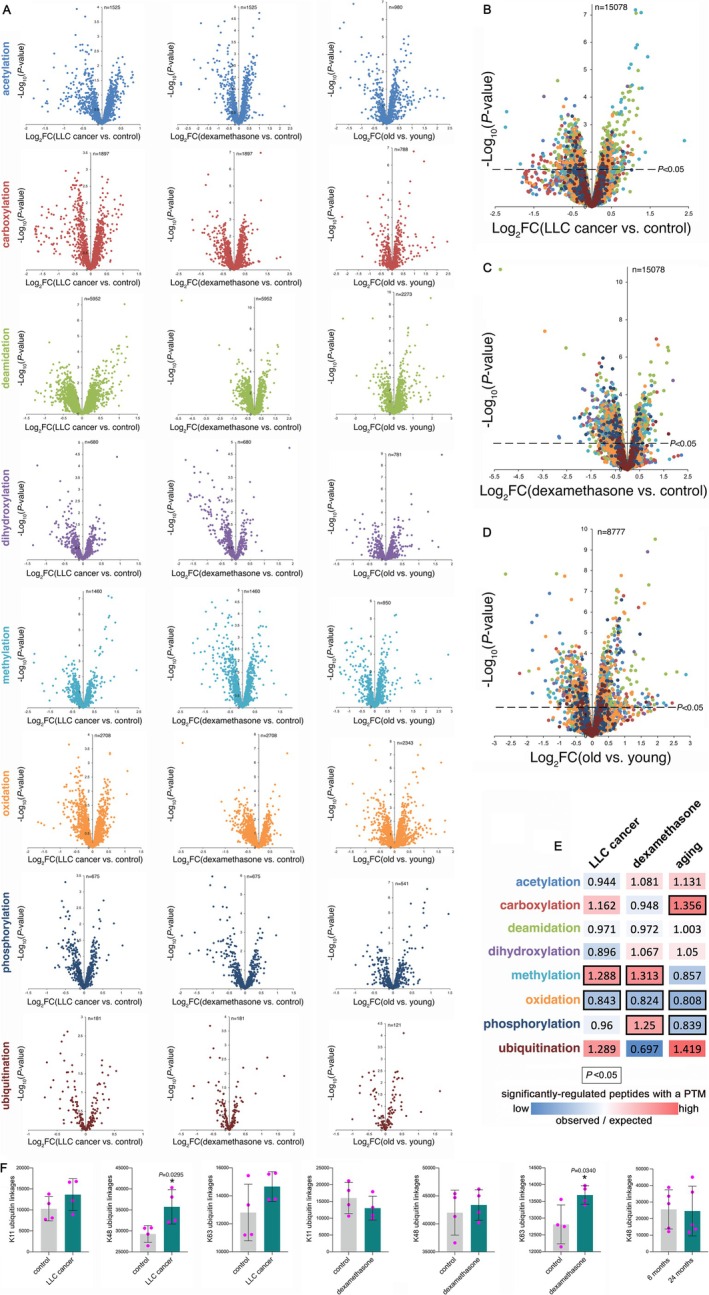
Post‐translational modifications associated with muscle wasting induced by cancer, dexamethasone and aging. (A) Identification of post‐translational modifications (PTMs) with JUMPptm from the TMT mass spectrometry of tibialis anterior (TA) skeletal muscle in which wasting is induced by cancer (LLC cancer cells vs. control; *n* = 4/group), dexamethasone treatment (*n* = 4/group) and aging (24 vs. 6 months; *n* = 5/group). Acetylation (blue), carboxylation (red), deamidation (green), dihydroxylation (purple), methylation (cyan), oxidation (orange), phosphorylation (navy blue) and ubiquitination (maroon) are differentially remodelled in TA muscles depending on the atrophic stimulus, i.e., cancer (B), dexamethasone (C) and aging (D). In (A) to (D), the number of detected PTMs is reported in each graph, with a total PTM number of *n* = 15 078 (cancer and dexamethasone) and *n* = 8777 (aging). The *x*‐axis reports the log_2_ fold changes (FC) in muscle wasting (cancer, dexamethasone and aging) vs. control, whereas the *y*‐axis indicates the significance, −log_10_ (*p* value). (E) Some PTMs in significantly regulated peptides are over‐represented compared to what is expected. The graph displays the proportion of observed versus expected peptides with PTMs that are significantly regulated (*p* < 0.05) when comparing LLC cancer vs. control, dexamethasone vs. control and 24 months (old) vs. 6 months (young). The expected number of significantly modified peptides with a specific PMT was estimated based on the prevalence of that PTM (Source data file). Lower‐than‐expected values are shown in blue, whereas higher values are shown in red. Outlined boxes indicate a significance of *p* < 0.05 (*χ* ^2^ test). Several PTMs are differentially associated with distinct modes of atrophy induction. Collectively, methylation and oxidation are significantly modulated by cancer cachexia and dexamethasone (which also significantly modulates phosphorylation), whereas carboxylation, oxidation and phosphorylation are associated with aging. (F) Changes in linkage‐specific ubiquitination are associated with muscle wasting induced by distinct stimuli. JUMPptm identifies linkage‐specific ubiquitination patterns, irrespective of the specific substrates. This analysis indicates that cancer cachexia significantly increases K48‐linked ubiquitination, whereas K63‐linked ubiquitination is up‐regulated by dexamethasone vs. controls. Apart for K48‐linked ubiquitination, other types of ubiquitin linkages were not detected for aging. The graphs report the mean ±SD, with *n* = 4 biological replicates (for cancer and dexamethasone) and *n* = 5 biological replicates (for aging); *p < 0.05, unpaired two‐tailed *t* test.

Because PTMs represent an important layer of proteome regulation, here we examined the PTMs that are commonly and distinctly regulated during muscle wasting induced by cancer, dexamethasone and aging. Specifically, we utilized the JUMPptm pipeline [13] to recover information on peptides that carry eight types of PTMs that are typically most common and/or of interest: acetylation, carboxylation, deamidation, dihydroxylation, methylation, oxidation, phosphorylation and ubiquitination (Figure 1A). Starting from enrichment‐free proteomic analyses generated via TMT mass spectrometry [12], JUMPptm identified peptides that carry PTMs because of their altered mass compared to the unmodified peptides (Figure 1A–D). Collectively, these analyses (Datasets S1 and S2 and Supplementary Information Figure S1) identified *n* = 15 078 PTMs for cancer (Figure 1B) and dexamethasone (Figure 1C) and *n* = 8777 PTMs for aging (Figure 1D) from the original TMT mass spectrometry datasets that profiled the proteomic changes that occur during muscle wasting induced by these triggers [12].

Analysis of the overall frequency indicates that some PTM types are over‐represented compared to the expected levels (Figure 1E). In particular, methylation is observed significantly more frequently than expected in muscle wasting induced by cancer and dexamethasone vs. controls, whereas oxidation is conversely regulated. Phosphorylation is significantly more recurrent in dexamethasone‐induced wasting but not in response to cancer and aging. Similar to cancer and dexamethasone‐induced wasting, sarcopenia (i.e., age‐induced decline in muscle mass and function) is characterized by a significantly lower‐than‐expected abundance of oxidative modifications, but also by a significant decline in phosphorylation, and higher observed than expected levels of peptides that are carboxylated (Figure 1E). Altogether, these surveys indicate a divergent abundance of different PTM types during muscle wasting induced by distinct atrophic stimuli.

Although many PTMs are modulated by atrophic stimuli, some of these PTMs are spontaneous and do not require any enzymatic reaction, as it is the case for deamidation. On this basis, we examined whether amino acid residues surrounding the modified site could influence such modifications. To this purpose, we examined the peptides that are significantly modified by each atrophic trigger and identified consensus sequences and amino acid residues that are statistically over‐ or under‐represented adjacent to the site modified by acetylation, carboxylation, deamidation, dihydroxylation, methylation, oxidation, phosphorylation and ubiquitination (Supplementary Information Figure S2 and Dataset S3). Interestingly, although the consensus sequences largely overlap, there are also stimulus‐specific differences that indicate a preferential bias in the modification of different proteins by distinct atrophic triggers (Supplementary Information Figure S2).

In addition to global changes in PTMs and PTM patterns, specific modifications were linked with muscle wasting induced by diverse atrophic stimuli. In particular, previous studies demonstrated a key role for protein ubiquitination in mediating the functional modifications and muscle mass proteolysis that leads to muscle wasting and weakness [4]. Poly‐ubiquitin chains can be assembled via the sequential linkage of ubiquitin tags, which typically occurs at lysine residues of ubiquitin [30]. Because poly‐ubiquitin chains can exert different functions depending on the lysine‐specific linkages of its ubiquitin components [30], we examined whether there is any difference in the pattern of linkage‐specific ubiquitination, irrespective of the specific protein substrates that are ubiquitinated [14]. To this purpose, we utilized JUMPptm to examine the ubiquitin peptides that are modified by ubiquitination and that, therefore, are indicative of the prevalent modes of poly‐ubiquitin chain assembly during muscle wasting.

These analyses indicate that cancer cachexia significantly increases ubiquitination at lysine 48 but not at other lysine residues of ubiquitin (K11 and K63) that are detected by JUMPptm, indicating that poly‐ubiquitin chains are preferentially assembled via K48 linkages during cancer‐induced muscle wasting (Figure 1F). Interestingly, dexamethasone significantly increases K63‐linked ubiquitination but not K11 and K48 ubiquitination. Lastly, K48‐linked ubiquitination does not significantly change with aging in skeletal muscle (Figure 1F). Therefore, these analyses identify a stimulus‐specific bias in the pattern of poly‐ubiquitin chain assembly that occurs during muscle wasting induced by different causes. Altogether, we identify a complex repertoire of modified peptides and PTM patterns during muscle wasting induced by aging, cancer and dexamethasone.

### 3.2 Largely Distinct Post‐Translational Modifications Differentiate Skeletal Muscle Wasting Induced by Cancer, Dexamethasone and Aging

**FIGURE 2:**
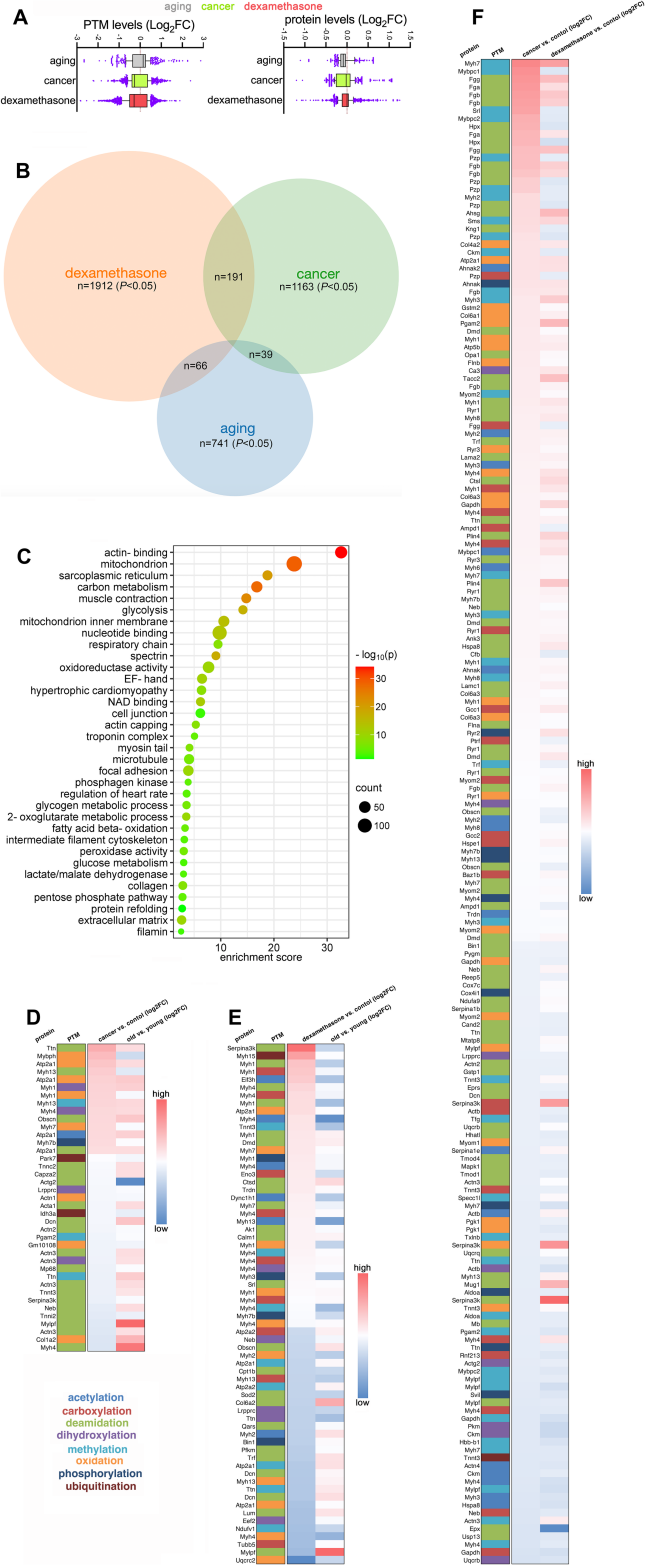
PTMs that are significantly regulated by aging, cancer and dexamethasone are largely stimulus‐specific. (A) Overview of the significant changes (*p* < 0.05) that occur in the levels of modified peptides (PTMs) and corresponding proteins in response to aging (grey), cancer (green) and dexamethasone (red) versus controls. The *y*‐axes report the log_2_ fold changes in peptide and protein levels; the median is shown together with whiskers corresponding to the 10–90 percentiles. (B) Venn diagrams that display the overlay between modified peptides that are significantly modulated (*p* < 0.05) by distinct atrophic stimuli, i.e., aging (grey), cancer (green) and dexamethasone (orange) versus controls. The number of PTMs for each group is indicated. Overall, most of the modified peptides are significantly modulated during muscle wasting by only a specific trigger, whereas relatively few are shared by > 1 atrophy inducers. (C) GO term analysis of the *n* = 600 proteins that carry one or more PTMs that are significantly modulated in response to aging, cancer and/or dexamethasone. The enrichment score, count and significance are shown for each category. (D–F) PTMs that are significantly and commonly regulated by aging and cancer (D), dexamethasone and aging (E) and dexamethasone and cancer (F). These shared PTMs are not necessarily regulated with the same magnitude and/or in the same direction. The log_2_ fold change of regulation is colour‐coded (with red indicating higher values and blue indicating lower values). Each type of PTM is colour‐coded as indicated.

Global analysis of the fold changes detected in all modified peptides indicates a significant difference between dexamethasone vs. cancer and dexamethasone vs. aging, which is also observed at the protein level (Figure 2A). The median levels of log_2_ fold changes in modified peptides increase with aging but overall decrease with dexamethasone and cancer vs. controls (Figure 2A). In line with differences in PTM changes depending on the atrophic stimulus, collective analyses of the prevalence of different types of PTMs (Figure 1E) and ubiquitin linkages (Figure 1F) further suggest that the PTMs that occur in skeletal muscle are largely distinct and depend on the specific atrophic stimulus.

To further refine this analysis, we next compared the overlap between the peptides that carry PTMs and that are significantly modulated (*p* < 0.05) during skeletal muscle wasting induced by cancer, dexamethasone and aging vs. controls (respectively, *n* = 1912, *n* = 1163 and *n* = 741). Although most PTM‐modified peptides are significantly modulated by only one intervention that induces atrophy (Figure 2B), there are small subsets of modified peptides that are commonly regulated (although not necessarily in the same direction) by aging and cancer (*n* = 39), aging and dexamethasone (*n* = 66) and cancer and dexamethasone (*n =* 191), whereas only *n* = 10 modified peptides are significantly modulated by all three atrophic stimuli (Figure 2B).

To gain a better understanding of the proteins that are impacted by these PTMs, we next compiled a non‐redundant list of proteins (*n* = 600) to which one or more significantly up‐regulated and down‐regulated peptides with PTMs are mapped (Figure 2C). Gene ontology analysis of proteins that carry significantly modulated PTMs indicates several categories that are enriched, including components of the muscle contractile apparatus (e.g., ‘actin‐binding,’ ‘troponin complex,’ ‘myosin tail’), mitochondria (e.g., ‘mitochondrion inner membrane,’ ‘respiratory chain’), metabolism (e.g., ‘glycolysis,’ ‘phosphagen kinase,’ ‘fatty acid beta‐oxidation’) and calcium handling (e.g., ‘sarcoplasmic reticulum,’ ‘EF‐hand domain’). Altogether, these analyses highlight the modulation of many protein categories necessary for muscle force production (Figure 2C).

Detailed analysis of the modified peptides that are consistently regulated by aging and cancer (Figure 2D), dexamethasone and aging (Figure 2E) and dexamethasone and cancer (Figure 2F) indicates that although these modified peptides are commonly regulated by two distinct atrophic stimuli, their magnitude and direction of regulation are not consistent. For example, deamidation of Ttn (titin) at N16419 increases in cancer vs. control but more so than what is observed during aging. Moreover, the oxidation of Mybph (myosin binding protein H) at P19 increases with cancer but decreases with aging (Figure 2D). Altogether, cross‐shared PTMs that are commonly regulated by more than one atrophic stimulus can be divergently regulated depending on the mode of muscle wasting.

### 3.3 Modified Peptides That Are Commonly Regulated by Multiple Atrophic Stimuli Include Lrpprc^P27^ Dihydroxylation

Our analyses identified modified peptides that are differentially regulated during muscle wasting induced by different triggers. Consistent with the analysis of proteomic changes [12], peptides modified by different PTMs are largely modulated by individual atrophic stimuli, with only a minority that is shared by more than two atrophy inducers, although not necessarily with the same magnitude and trajectory of regulation. Therefore, these findings support a model whereby muscle wasting can be induced via multiple different changes at the proteome and PTM level and that these changes are largely stimulus‐specific. However, our proteomic analyses have identified a few protein markers (atroproteins) that are commonly, if not generally, induced during muscle wasting. These include Ctsl (cathepsin L), which is consistently induced at the mRNA and protein levels by cancer, dexamethasone and aging [12]. On this basis, we next sought to define PTMs that are commonly and consistently regulated by multiple atrophic stimuli and that therefore may represent widespread, if not general, markers of muscle wasting.

**FIGURE 3:**
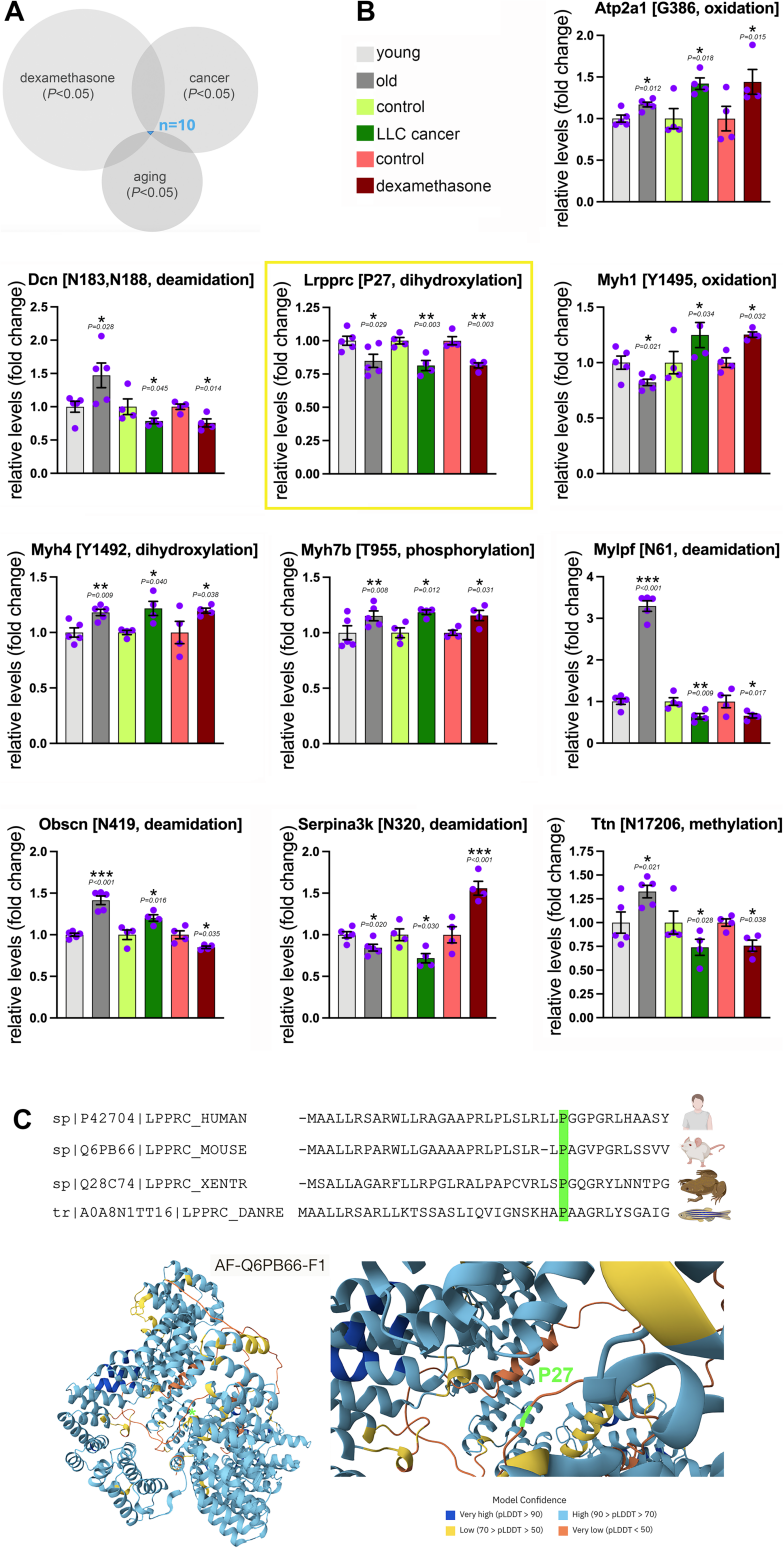
Cross‐shared PTMs that are significantly regulated by aging, cancer and dexamethasone include a consistent down‐regulation of Lrpprc dihydroxylation at P27. (A) Only 10 PTMs are significantly (*p* < 0.05) cross‐regulated by aging, cancer and dexamethasone versus their own respective controls. (B) Of these, six PTMs are discordantly regulated, and only four PTMs are significantly and consistently regulated by aging, cancer and dexamethasone versus the associated controls: Serca1/Atp2a1^G386^ oxidation, Myh7b^T955^ phosphorylation and Myh4^Y1492^ dihydroxylation (which increase in atrophic conditions) and Lrpprc^P27^ dihydroxylation (which decreases in muscle wasting vs. control conditions). The graphs display the mean ± SEM and *n* = 5 (aging) and *n* = 4 (cancer, dexamethasone). *p* values related to JUMPptm analyses were calculated with a moderated *t* test; *p < 0.05, **p < 0.01, ***p < 0.001. (C) Evolutionary conservation of proline 27 in Lrpprc from different species. Alignment of the Lrpprc (leucine‐rich pentatricopeptide repeat containing) protein sequences from human (sp|P42704|LPPRC_HUMAN), mouse (sp|Q6PB66|LPPRC_MOUSE), *Xenopus* (sp|Q28C74|LPPRC_XENTR) and zebrafish (tr|A0A8N1TT16|LPPRC_DANRE) indicates that P27 is conserved across diverse animal species. The protein structure of mouse Lrpprc (as predicted by AlphaFold 3) indicates that P27 is located in an unstructured region.

To address this question, we examined the set of 10 PTMs that are cross‐shared in the three models of muscle wasting (Figure 3A). These modifications occur in peptides of contractile, leucine‐rich and other proteins and consist of oxidation of Atp2a1 (ATPase sarcoplasmic/endoplasmic reticulum Ca^2+^ transporting 1, also known as Serca1) at G386, deamidation of Dcn (decorin, a small leucine‐rich proteoglycan family protein) at N183‐N188, dihydroxylation of Lrpprc at P27, oxidation of myosin heavy chain 1 at Y1495, dihydroxylation of myosin heavy chain 4 at Y1492, phosphorylation of myosin heavy chain 7B at T955, deamidation of Mylpf (myosin light chain 11) at N61, deamidation of the sarcomeric protein obscurin at N419, deamidation of the protease Serpina3k at N320 and methylation of titin at N17206 (Figure 3B). Only four of these modified peptides are significantly and consistently regulated in the same direction by aging, cancer and dexamethasone: Atp2a1^G386^ oxidation, Myh7b^T955^ phosphorylation and Myh4^Y1492^ dihydroxylation increase in atrophic vs. control conditions, whereas Lrpprc^P27^ dihydroxylation decreases in muscle wasting vs. controls (Figure 3B). Therefore, these analyses suggest that a few modified peptides are potentially widespread, if not universal, markers of muscle wasting irrespective of the inducing trigger.

LRPPRC, also known as Lrp130, is a mRNA binding protein and transcriptional coactivator that regulates mitochondrial function and is mutated in patients with French–Canadian Leigh syndrome [16, 17, 18]. Although Lrpprc protein (but nor mRNA) levels decline in response to aging and dexamethasone treatment, this does not occur following cancer cachexia (Supplementary Information Figure S3).

Consultation of the AlphaFold structure prediction indicates that P27 is located within an unstructured region (Figure 3C). Although no mutation in proline 27 has been found in Leigh syndrome patients, proline 27 is evolutionarily conserved in human, mouse, *Xenopus* and zebrafish LRPPRC (Figure 3C), suggesting that this amino acid residue is functionally important. Altogether, these analyses identify Lrpprc^P27^ dihydroxylation as a biomarker of muscle wasting.

### 3.4 Experimental Impediment of Lrpprc^P27^ Dihydroxylation Reduces Muscle Strength But Not Muscle Mass in Young Mice

Our analyses identified modified peptides that are significantly modulated by single inducers of muscle atrophy. Only a few are cross‐shared (~universal) markers induced by multiple triggers of atrophy, including a decline in Lrpprc^P27^ dihydroxylation. Considering the important role of Lrpprc in human disease [16, 17, 18], we reasoned that Lrpprc^P27^ dihydroxylation may not merely represent a biomarker of muscle wasting but also functionally contribute to it. To address this hypothesis, we utilized our established protocols for the in situ measurement of force production by the TA muscle [23, 25]. For these studies, we electroporated contralateral TA muscles of each mouse with either a plasmid for the expression of Flag‐tagged wild‐type Lrpprc (Lrpprc^WT^) or with a plasmid that encodes for Flag‐tagged Lrpprc with a P27A mutation that makes it refractory to hydroxylation (Lrpprc^P27A^). In support of this strategy, it was previously shown that proline can be substituted by alanine to obtain a proline‐hydroxylation–resistant variant. For example, this approach was previously utilized to demonstrate that hydroxylation of the DYRK1 kinase at P332 is required for its kinase activity [24].

**FIGURE 4:**
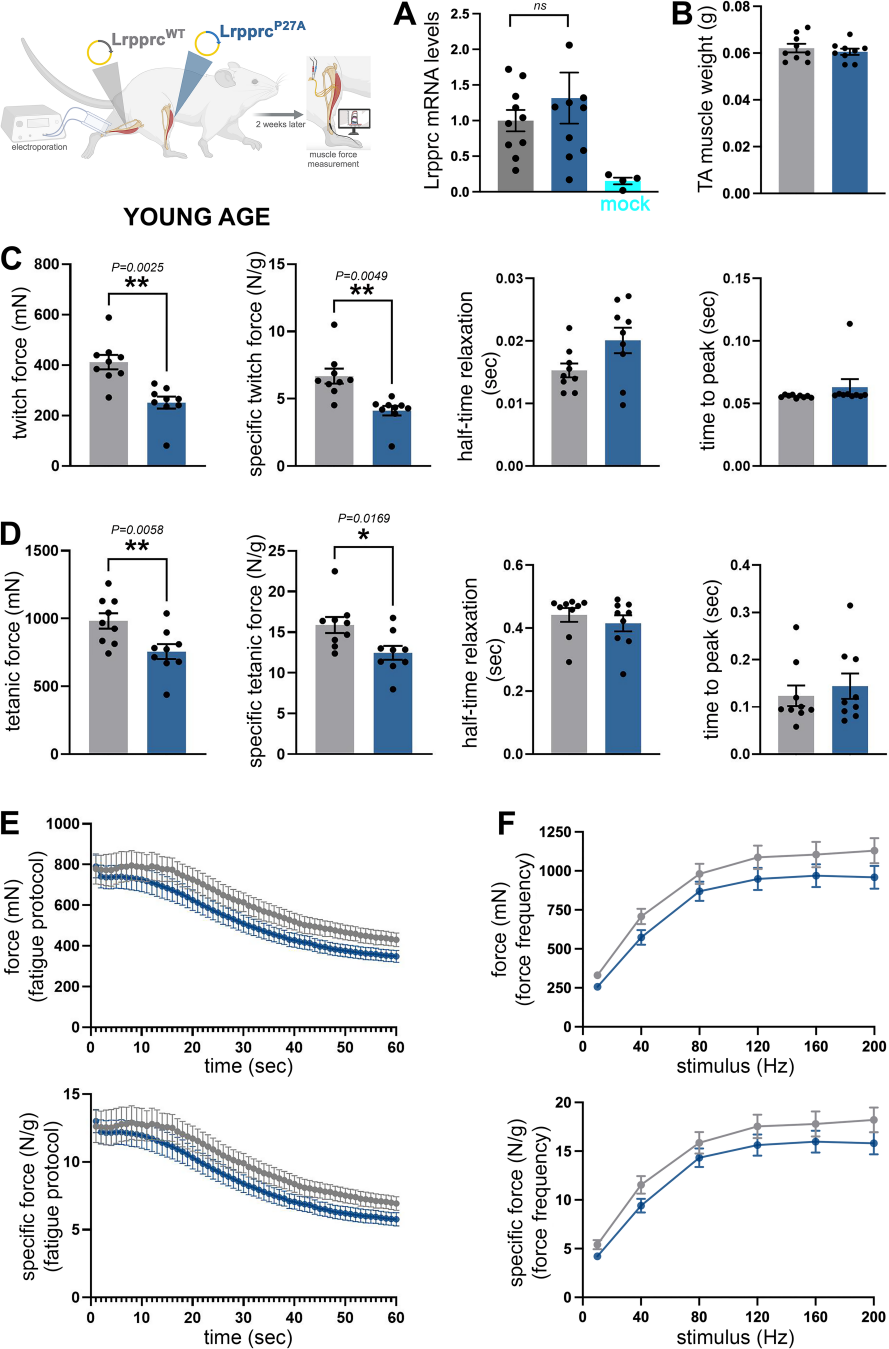
Modulation of muscle force by Lrpprc^P27A^ vs. Lrpprc^WT^ in young mice. Dihydroxylation of Lrpprc at P27 declines in response to atrophic stimuli, and its P27A mutation (which prevents hydroxylation) is utilized to mimic the decline in Lrpprc^P27^ dihydroxylation that occurs with muscle wasting. For these experiments, Lrpprc^WT^ (grey) and Lrpprc^P27A^ (blue) were electroporated into contralateral tibialis anterior muscles, and the muscle force was analysed 2 weeks later. (A) qRT‐PCR indicates that similar *Lrpprc* mRNA levels are detected following the electroporation of Lrpprc^P27A^ versus Lrpprc^WT^ into contralateral TA muscles (*n* = 9) and that these are higher compared to mock‐electroporated TA muscles (*n* = 4). The graph displays the mean ± SEM (B) Lrpprc^P27A^ does not change the mass of the tibialis anterior (TA) muscle versus Lrpprc^WT^. (C) Muscle force measurement indicates that Lrpprc^P27A^ reduces the twitch force and the normalized twitch force, with no significant changes in the half‐time relaxation and the time to peak. (D) Similar results were also found from measuring the tetanic force, which decreases in Lrpprc^P27A^ muscles versus Lrpprc^WT^ muscles. Although there was a trend toward reduced force, there was no significant change in the fatigue protocol (E) and in the force–frequency (F). The graphs display the mean ± SEM and *n* = 9. *p* values were calculated with a paired *t* test; *p < 0.05, **p < 0.01.

Comparison of contralateral TA muscles from young (4‐month‐old) male mice electroporated with Lrpprc^P27A^ vs. Lrpprc^WT^ indicates that these electroporated variants are similarly expressed compared to mock‐electroporated control TA muscles (Figure 4A). Although there is no change in muscle mass (Figure 4B), a significant decline in the twitch force is observed in Lrpprc^P27A^ vs. Lrpprc^WT^ muscles also when normalized by the muscle weight (Figure 4C) and that this occurs without changes in the half‐time of relaxation and the time to peak (Figure 4C). The tetanic force and the specific tetanic force are also significantly reduced by Lrpprc^P27A^ vs. Lrpprc^WT^ with no changes in other contractile properties (Figure 4D). Lastly, there are no significant differences in fatigue (Figure 4E) and force–frequency (Figure 4F), although Lrpprc^P27A^ displays lower values than Lrpprc^WT^. Altogether, these findings indicate that a dihydroxylation‐resistant Lrpprc variant (Lrpprc^P27A^, which mimics the decline in Lrpprc^P27^ dihydroxylation that occurs during muscle wasting) reduces muscle force production in young mice compared to Lrpprc^WT^.

We also tested whether Lrpprc up‐regulation impacts muscle strength compared to GFP controls and found that this is not the case. Specifically, the TA muscle mass, the twitch force, the tetanic force and the associated contractile properties (half‐time relaxation and time to peak) are not significantly regulated when comparing TA muscles (from young mice) electroporated with GFP vs. TA muscles (from the experiment above) electroporated with Lrpprc^WT^ (Supplementary Information Figure S4).

### 3.5 The Dihydroxylation‐Resistant Lrpprc^P27A^ Variant Does Not Impact Myofibre Size, Type and Number Compared to Wild‐Type Lrpprc^WT^ in Young Mice

**FIGURE 5:**
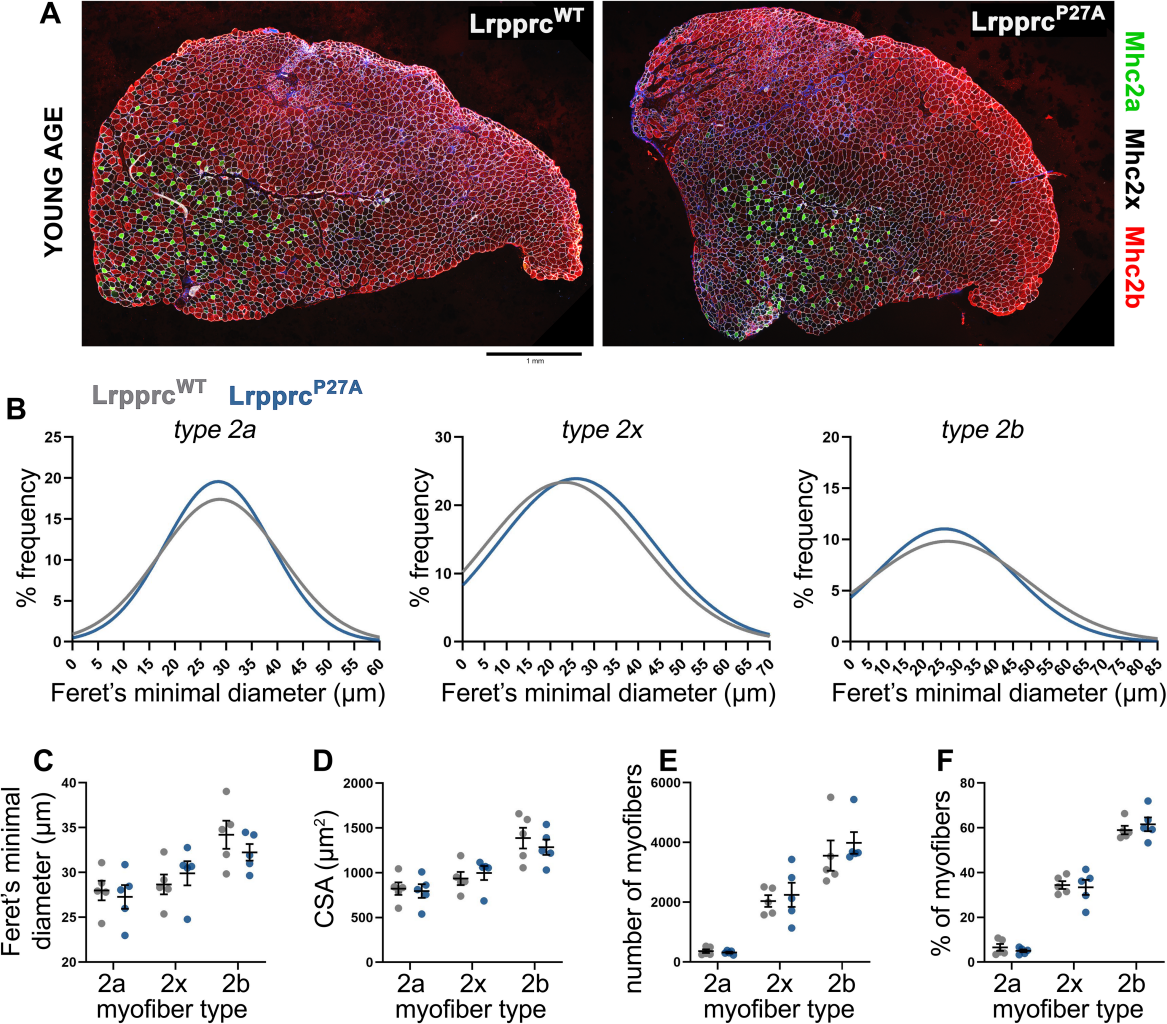
Lrpprc^P27A^ does not regulate myofibre size, type or number in young mice. (A) Immunostaining and confocal microscopy of transverse sections of contralateral tibialis anterior (TA) skeletal muscles electroporated with Lrpprc^P27A^ vs. Lrpprc^WT^. Immunoreactivity to myosin heavy chain isoforms is utilized to define Type 2a (Mhc2a‐positive; green), Type 2b (Mhc2b‐positive; red) and Type 2x (Mhc2a/Mhc2b‐negative; black) myofibres. Staining for laminin α2 (white) is utilized to define the myofibre boundaries. (B–D) Gaussian distributions (B) and frequency (C) of the Feret's minimal diameters for Types 2a, 2x and 2b myofibres indicate that there is no significant difference in the size of Types 2a, 2x and 2b myofibres, as also indicated by the analysis of the cross‐sectional area, CSA (D). (E and F) The number of myofibres (E) and the relative proportion of myofibre types (F) is also not affected by Lrpprc^P27A^ compared to Lrpprc^WT^. The graphs display the mean ± SEM from *n* = 5 biological replicates (muscles) per group, obtained from independent mice. Statistical analysis was done with two‐way ANOVA.

A universal component of muscle wasting is the decline in myofibre size. Although we did not observe any change in muscle mass in response to electroporation of the dihydroxylation‐resistant Lrpprc^P27A^ variant compared to Lrpprc^WT^ (Figure 4B), we hypothesized that there might be changes in myofibre size that are relatively minor and that therefore do not lead to obvious changes in muscle mass. To test this hypothesis, transverse cross‐sections of contralateral TA muscles (from young mice) electroporated with Lrpprc^P27A^ vs. Lrpprc^WT^ were immunostained with antibodies specific for different myosin heavy chain isoforms (Figure 5A). In addition to estimating the size of different myofibre types (Figure 5B–D), we also examined the number and percentage of myofibre types (Figure 5E–F), although these parameters are not commonly impacted during muscle wasting [4].

Immunostaining, confocal microscopy and image analysis revealed that the size (Figure 5B–D), number (Figure 5E) and relative proportion (Figure 5F) of Types 2a, 2x and 2b myofibres are not significantly affected by Lrpprc^P27A^ vs. Lrpprc^WT^. Altogether, these analyses indicate that the dihydroxylation‐resistant Lrpprc^P27A^ variant does not induce myofibre atrophy (Figure 5). Moreover, the average myofibre size and the myofibre number were also not affected when comparing TA muscles electroporated with Lrpprc^WT^ vs. GFP (Supplementary Information Figure S5A–C).

Taken together with the finding that Lrpprc^P27A^ impairs muscle force production compared to Lrpprc^WT^ (Figure 4), these results indicate that the atrophy‐associated decline in Lrpprc dihydroxylation may contribute to disease‐associated muscle weakness via mechanisms other than myofibre atrophy.

### 3.6 Lrpprc P27 Dihydroxylation Is Dispensable for Mitochondrial Function

Regulation of mitochondrial function is one of the most prominent roles that Lrpprc plays in physiological conditions [19, 20, 21]. On this basis, we next examined whether mitochondrial function is perturbed by Lrpprc^P27A^ vs. Lrpprc^WT^. To this purpose, myofibres were isolated from TA muscles (from young mice) electroporated with Lrpprc^P27A^ and Lrpprc^WT^. Subsequently, the Seahorse XF mito stress assay was utilized to measure the oxygen consumption rate of Lrpprc^P27A^ vs. Lrpprc^WT^‐expressing myofibres following the administration of oligomycin (an inhibitor of Complex V), FCCP (a mitochondrial uncoupler used to estimate the maximal respiration) and rotenone (an inhibitor of Complex I) plus antimycin A (an inhibitor of Complex III). Similar to other Seahorse profiles obtained from tissues or tissue‐derived cells [28, 29], there were relatively small responses to these mitochondrial modulators compared to cell‐based studies. Moreover, as estimated with this assay, there were no substantial differences in the mitochondrial function of Lrpprc^P27A^ vs. Lrpprc^WT^ myofibres (Supplementary Information Figure S6A).

Previous studies found that Lrpprc is located in mitochondria, where it controls the stability of mitochondrial mRNAs and the assembly of Complex IV of the electron transport chain [19, 20, 21], which is perturbed in Leigh syndrome [16, 17, 18], where defects in Complex I are also observed [31]. To test whether Lrpprc P27 dihydroxylation has a role in this process, we utilized an assay that specifically measures the activity of Complex I and Complex IV. However, also in this case, there was no difference in Complex I and Complex IV activity when comparing TA muscles electroporated with Lrpprc^P27A^ vs. Lrpprc^WT^. In summary, these findings indicate that preventing Lrpprc P27 dihydroxylation does not seem to impact mitochondrial function (Supplementary Information Figure S6B).

Defective assembly of Complex IV is a typical outcome of disease‐causing mutations in Lrpprc [19, 20, 21]; however, defects in the assembly of Complex I have also been reported [18]. On this basis, we monitored the assembly status of different complexes of the electron transport chain by utilising a set of antibodies that recognize labile complex subunits (i.e., NDUFB8, SDHB, UQCRC2, MTCO1 and ATP5A) that are stable only when part of Complex I, II, III, IV and V, respectively. These analyses revealed no significant changes in the assembly of mitochondrial complexes I, II, III, IV and V when comparing muscle homogenates from TA muscles electroporated with Lrpprc^P27A^ vs. Lrpprc^WT^ (Supplementary Information Figure S6C). Lastly, we examined the levels of the transcription factor PGC‐1α, a master regulator of mitochondrial biogenesis that was previously found to functionally interact with Lrpprc [32], but found no change in PGC‐1α protein levels in TA skeletal muscles that express Lrpprc^P27A^ vs. Lrpprc^WT^ (Supplementary Information Figure S6D). Altogether, these studies indicate that Lrpprc P27 dihydroxylation is dispensable for several aspects of mitochondrial function.

We also compared TA muscles electroporated with Lrpprc^WT^ vs. control GFP but found no significant changes in the assembly status of the five complexes of the electron transport chain (Supplementary Information Figure S7), suggesting that Lrpprc up‐regulation does not impact mitochondrial function.

### 3.7 Lrpprc P27 Dihydroxylation Does Not Impact Proteostasis

Loss of proteostasis has emerged as an important component of sarcopenia [6, 23]. A decline in the function of proteolytic systems (in particular, the ubiquitin–proteasome and autophagy–lysosome) that degrade damaged proteins and organelles can lead to the accumulation of insoluble poly‐ubiquitinated proteins and dysfunctional organelles with aging [6, 23]. Conversely, activation of proteolytic systems sustains the rapid muscle mass loss that occurs during atrophy in the young [4]. Considering the important roles of proteostasis in muscle wasting, we next examined whether this process is differentially regulated in muscles electroporated with Lrpprc^P27A^ vs. Lrpprc^WT^. To this purpose, we monitored the levels of poly‐ubiquitinated proteins but found no significant changes in Lrpprc^P27A^ vs. Lrpprc^WT^ muscles (Supplementary Information Figure S8). Next, we investigated whether macroautophagy is regulated by assessing the levels of LC3, a cytoplasmic protein (LC3‐I) which is lipidated (LC3‐II) to form new autophagosomes. In addition, we also monitored the levels of phospho‐Atg16 (pATG16L1), which is a reliable indicator of the autophagic rate because it marks only newly forming autophagosomes. These analyses indicated that there is no significant change in the levels of phospho‐Atg16, LC3‐I, LC3‐II and the LC3‐II/LC3‐I ratio (Supplementary Information Figure S8), indicating that autophagy is not regulated by Lrpprc^P27A^ vs. Lrpprc^WT^ in skeletal muscle.

Likewise, there were no changes in the levels of these proteostasis markers when comparing Lrpprc^WT^ vs. GFP (Supplementary Information Figure S9).

### 3.8 Lrpprc^P27A^ Perturbs the Levels of Key mRNAs Necessary for Muscle Force Production

**FIGURE 6:**
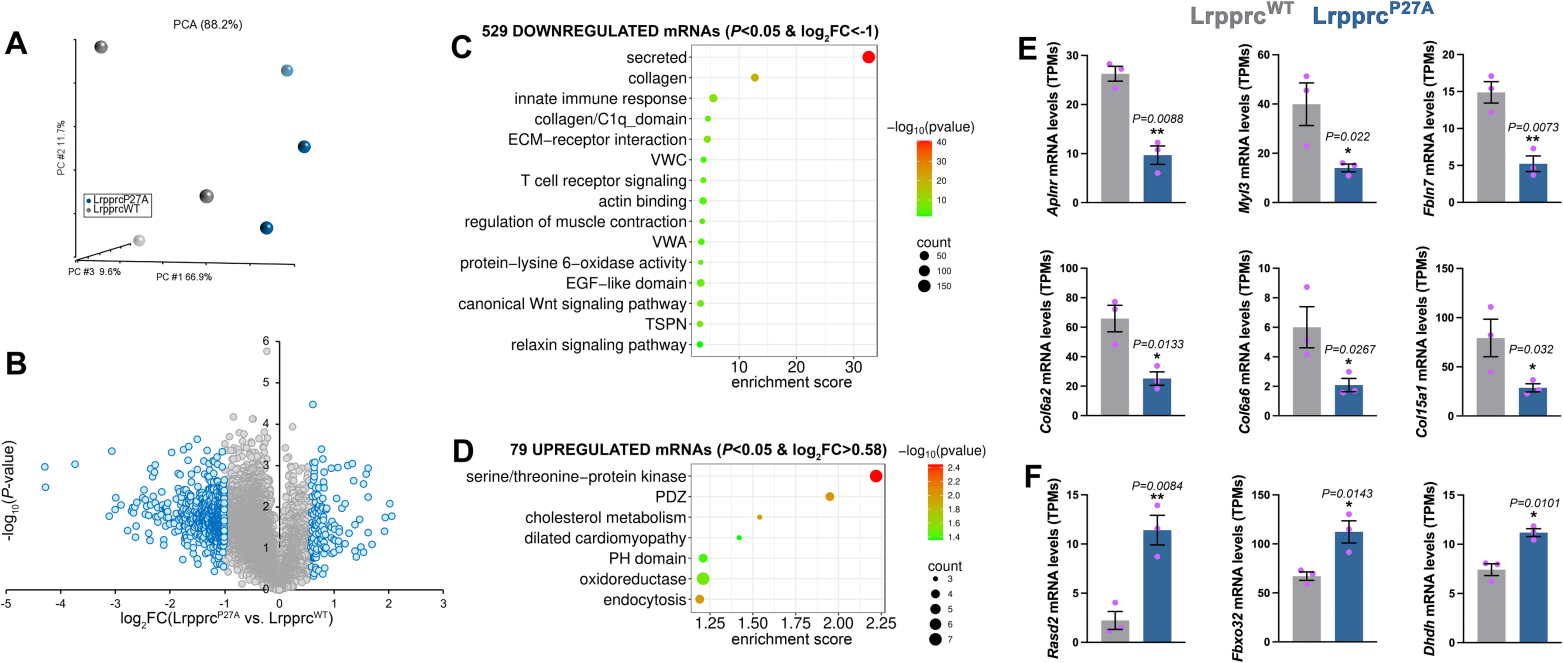
Lrpprc^P27A^ impairs the expression of genes necessary for muscle strength. RNA‐seq of the transcriptional changes induced by Lrpprc^P27A^ vs. Lrpprc^WT^ in contralateral TA muscles. (A) Principal component analysis (PCA) indicates the clustering of Lrpprc^WT^ (grey) and Lrpprc^P27A^ (blue) samples (*n* = 3 biological replicates/group). (B) Overview of the transcriptional changes indicates that several genes are down‐regulated by Lrpprc^P27A^ vs. Lrpprc^WT^, whereas fewer are up‐regulated. The *x*‐axis reports the log_2_FC (fold change) of Lrpprc^P27A^ versus Lrpprc^WT^, whereas the *y*‐axis indicates the −log_10_ (*p* value). (C and D) GO terms that are enriched among differentially regulated genes (529 down‐regulated genes with *p* < 0.05 and log_2_FC < −1 [C] and 79 up‐regulated genes with *p* < 0.05 and log_2_FC > 0.58 [D]). The enrichment score, count and −log_10_ (*p* value) are indicated. (E and F) Representative genes that are significantly (*p* < 0.05) down‐regulated (E) and up‐regulated (F) by Lrpprc^P27A^ versus Lrpprc^WT^ include regulators of muscle homeostasis, muscle contraction and myopathies. The graphs display the mean ± SEM and *n* = 3. *p* values were calculated with an unpaired two‐tailed *t* test; *p < 0.05, **p < 0.01. Supplementary Information Figure S10 reports the modulation of these genes in response to aging, cancer and dexamethasone.

It was previously reported that Lrpprc is a coactivator that modulates the transcriptional responses induced by PGC‐1α and potentially by other transcription factors [32]. On this basis, we next examined whether there are gene expression changes that are modulated by Lrpprc^P27A^ vs. Lrpprc^WT^. RNA‐seq from Lrpprc^P27A^ vs. Lrpprc^WT^ TA muscles clustered separately (Figure 6A,B and Dataset S4) and revealed that Lrpprc^P27A^ reduces the levels of many categories compared to Lrpprc^WT^ (secreted factors, collagen, regulators of innate immune responses; Figure 6C), whereas relatively fewer mRNAs are up‐regulated (kinases, PDZ domain‐containing proteins, regulators of cholesterol metabolism; Figure 6D).

The mRNAs that are down‐regulated by Lrpprc^P27A^ vs. Lrpprc^WT^ encode for several known regulators of muscle strength and homeostasis, such as the *Aplnr* receptor for Apelin (a myokine that contrasts wasting), muscle contractile proteins such as *Myl3* (*myosin light chain 3*) and the cell adhesion protein *Fibulin 7* (*Fbln7*). Moreover, *Col6a2*, *Col6a6* and *Col15a1* collagens are down‐regulated by Lrpprc^P27A^ vs. Lrpprc^WT^ (Figure 6E) but also have decreased expression during muscle wasting, albeit to different degrees depending on the atrophic stimulus (Supplementary Information Figure S10). Interestingly, *Col6a2* and *Col6a6* are mutated in Bethlem myopathy and Ullrich muscular dystrophy [33], diseases characterized by muscle strength decline, suggesting that lower *Col6a* and *Col6a6* expression caused by Lrpprc^P27A^ may contribute to the reduction in muscle force caused by Lrpprc^P27A^ and observed during atrophy. Consistent with this idea, *Col6a2* knockout was previously reported to reduce muscle force, at least in part by modulating TGFβ availability [34].

Lastly, mRNAs that are up‐regulated by Lrpprc^P27A^ vs. Lrpprc^WT^ include *Fbxo32*, also known as atrogin‐1, an E3 ubiquitin ligase that is transcriptionally up‐regulated by cancer cachexia and dexamethasone but not by aging [4, 12] (Supplementary Information Figure S10), the E3 ligase *Rasd2* (also known as *Rhes*), involved in sumoylation, and the enzyme *dihydrodiol dehydrogenase* (*Dhdh*), which contributes to xenobiotic metabolism (Figure 6F). Altogether, these analyses indicate that Lrpprc^P27A^ perturbs the mRNA levels of key proteins necessary for muscle strength and homeostasis.

### 3.9 Lrpprc^P27A^ Reduces Muscle Strength in Old Mice Compared to Lrpprc^WT^

We have found that electroporation of the dihydroxylation‐resistant Lrpprc^P27A^ variant reduces muscle strength compared to Lrpprc^WT^ (Figure 4). These studies were done in young (4‐month‐old) mice to test whether muscle force is impacted by mimicking the decline in Lrpprc dihydroxylation at P27 that occurs in response to cancer, dexamethasone and aging. However, muscle strength might be more profoundly affected if the atrophy‐associated decline in P27 Lrpprc dihydroxylation is further reduced experimentally in the context of atrophy.

**FIGURE 7:**
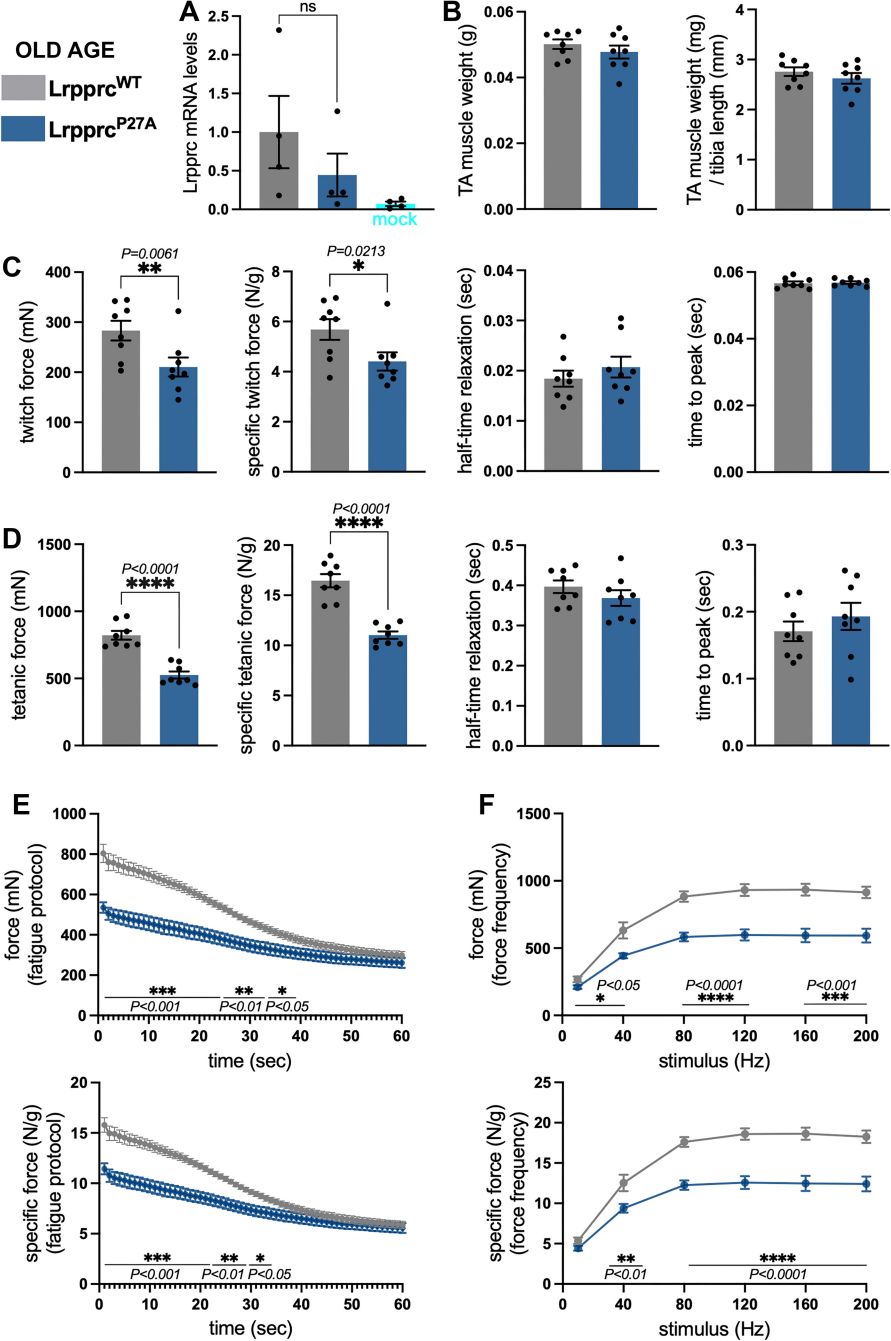
**Electroporation of Lrpprc** ^**P27A**^ **reduces muscle strength in old mice compared to Lrpprc** ^**WT**^. Electroporation of DNA plasmids for the expression of Lrpprc^WT^ (grey) and Lrpprc^P27A^ (blue) in contralateral TA muscles of 27‐month‐old male mice (*n* = 8). (A) qRT‐PCR indicates that there are no significant changes in *Lrpprc* mRNA levels when comparing TA muscles electroporated with Lrpprc^P27A^ vs. Lrpprc^WT^, but Lrpprc‐electroporated muscles have higher Lrpprc mRNA levels compared to mock‐electroporated muscles. The graph displays the mean ± SEM and *n* = 4. *p* values were calculated with the paired two‐tailed *t* test. (B) There is no significant change in the TA mass when comparing Lrpprc^P27A^ vs. Lrpprc^WT^, also when the TA muscle mass is normalized by the length of the tibia bone. (C) Lrpprc^P27A^ significantly reduces the twitch force compared to control muscles that express Lrpprc^WT^, also when normalized by the TA mass (specific twitch force). There are no significant changes in the half‐time relaxation and time to peak. (D) Lrpprc^P27A^ significantly reduces the tetanic force and specific tetanic force compared to control muscles that express Lrpprc^WT^, with no changes in the half‐time relaxation and time to peak. The graphs display the mean ± SEM with *n* = 8 biological replicates, and *p* values were calculated with the paired two‐tailed *t* test (*p < 0.05, **p < 0.01, ****p < 0.0001). (E and F) Lrpprc^P27A^ significantly reduces muscle force when assessed with the fatigue (E) and force–frequency (F) protocols, also when normalized by the TA mass (specific force). The graphs display the mean ± SEM with *n* = 7 (E) and *n* = 8 (F) biological replicates, and two‐way ANOVA was used for the statistical analysis (*p < 0.05, **p < 0.01, ***p < 0.001, ****p < 0.0001).

To test this hypothesis, we electroporated the dihydroxylation‐resistant Lrpprc^P27A^ variant in TA muscles of geriatric (27‐month‐old) male mice and compared the resulting outcome on muscle force to electroporation of Lrpprc^WT^ in contralateral TA muscles of the same mice. Analysis by qRT‐PCR indicates no significant differences in *Lrpprc* mRNA levels when comparing Lrpprc^P27A^ vs. Lrpprc^WT^ muscles (Figure 7A). As observed in young age (Figure 4), Lrpprc^P27A^ did not impact muscle mass compared to Lrpprc^WT^ (Figure 7B). However, Lrpprc^P27A^ significantly reduced the twitch force (Figure 7C) and the tetanic force (Figure 7D) also when normalized by the muscle mass (specific force), without affecting other parameters of muscle contraction (half‐time relaxation and time to peak). Although there were no significant changes in muscle fatigue and force–frequency in young age (Figure 4), Lrpprc^P27A^ electroporation in old age profoundly reduced muscle force compared to Lrpprc^WT^ when assessed with the fatigue (Figure 7E) and force–frequency (Figure 7F) protocols. We also examined whether Lrpprc^WT^ modulates muscle force compared to GFP electroporation in 27‐month‐old mice but found minimal effects (Supplementary Information Figure S11). Taken together, these findings indicate that experimental reduction of Lrpprc dihydroxylation at P27 (i.e., Lrpprc^P27A^) causes muscle weakness in young (Figure 4) and old age (Figure 7) but that the decline in muscle force resulting from this intervention is more pronounced in old age.

### 3.10 Lrpprc^P27A^ Reduces the Size of Type 2b Myofibres in Old Mice Compared to Lrpprc^WT^

**FIGURE 8:**
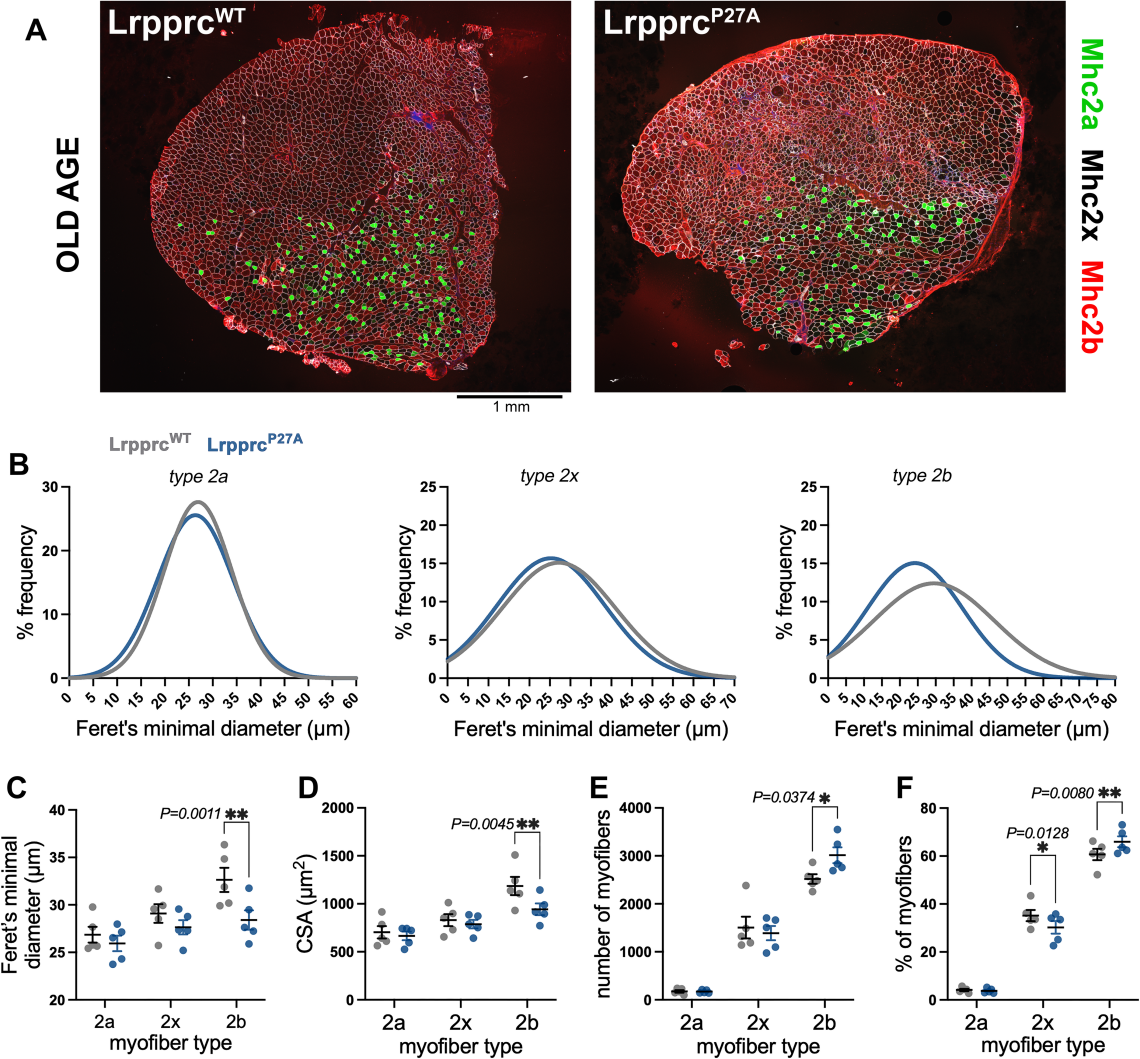
Lrpprc^P27A^ vs. Lrpprc^WT^ reduces myofibre size in old mice. (A) Immunostaining and confocal microscopy of contralateral tibialis anterior (TA) skeletal muscles electroporated with Lrpprc^P27A^ vs. Lrpprc^WT^ in 27‐month‐old male mice (*n* = 5). Immunoreactivity to myosin heavy chain isoforms identifies Type 2a (Mhc2a‐positive; green), Type 2b (Mhc2b‐positive; red) and Type 2x (Mhc2a/Mhc2b‐negative; black) myofibres. Staining for laminin α2 (white) is utilized to define the myofibre boundaries. (B‐D) The Gaussian distributions (B) and frequency (C) of the Feret's minimal diameters for Types 2a, 2x and 2b myofibres indicate that there is a significant difference in the size of Type 2b myofibres (but not of Types 2a and 2x), as also indicated by the analysis of the cross‐sectional area, CSA (D). (E and F) The number of myofibres (E) and the relative proportion of myofibre types (F) are also affected by Lrpprc^P27A^ compared to Lrpprc^WT^. The graphs display the mean ± SEM from *n* = 5 biological replicates (muscles) per group. The statistical analysis was done with two‐way ANOVA with Sidak's multiple comparison test (*p < 0.05, **p < 0.01).

Although myofibre size was not affected in young age (Figure 5), we hypothesized that the profound reduction in muscle strength caused by Lrpprc^P27A^ in 27‐month‐old mice could arise at least in part from myofibre atrophy. To test this hypothesis, transverse sections of contralateral TA muscles electroporated with either Lrpprc^P27A^ or Lrpprc^WT^ were immunostained with antibodies for myosin heavy chain isoforms to identify Types 2a, 2x and 2b myofibres and with anti‐laminin α2 antibodies to delineate the myofibre boundaries (Figure 8A). Analysis of the Gaussian plots indicates that there is an overall decline in the size of Type 2b myofibres in Lrpprc^P27A^ vs. Lrpprc^WT^ muscles and that this does not occur for 2a and 2x myofibres (Figure 8B). Analysis of the Feret's minimal diameter and the cross‐sectional area (CSA) further confirmed that Lrpprc^P27A^ causes a significant decline (~13%) in the size of 2b myofibres, compared to Lrpprc^WT^, and that this does not occur for Types 2a and 2x myofibres (Figure 8C,D). The decline in Type 2b myofibre size was accompanied by an increase in the total number and relative proportion of these myofibres (Figure 8E,F). In contrast, the average myofibre size and number were not affected when comparing TA muscles electroporated with Lrpprc^WT^ vs. GFP (Supplementary Information Figure S5D–F).

We also examined whether the assembly of oxphos complexes (Supplementary Information Figure S12) and the levels of proteostasis markers (Supplementary Information Figure S13) but found that these are not significantly affected in Lrpprc^P27A^ vs. Lrpprc^WT^ muscles in old age, as also found from the analyses in young age (Supplementary Information Figures S6 and S8).

Altogether, these analyses indicate that Lrpprc^P27A^ impacts muscle strength more profoundly in old vs. young age at least in part because of atrophy of Type 2b myofibres.

## 4 Discussion

Skeletal muscle wasting is a common co‐morbidity of many diseases, including cancer, but the underlying mechanisms are incompletely understood [4]. A general model of decreased protein synthesis and increased protein degradation is widely accepted to explain muscle wasting induced by multiple stimuli [4]. Genes transcriptionally induced by multiple atrophic stimuli (atrogenes) are a defining component of this model [4]. However, there are cellular and tissue discrepancies in the manifestation of wasting depending on the inducing trigger [4]. Moreover, the increasing availability of proteomic datasets on muscle wasting indicates a more complex scenario whereby muscle atrophy is defined by unique protein changes (atroproteins) that are largely stimulus‐specific and at least in part disconnected from mRNA changes [12].

Many of the changes in protein abundance and function that occur during muscle wasting have been ascribed to the increase in protein ubiquitination and consequent degradation [4]. However, other PTMs apart from ubiquitination are also modulated during muscle wasting and may have important functional roles. For example, PTMs of sarcomeric proteins (e.g., myosin heavy chains) occur during sarcopenia [35] and were found to regulate myofibril assembly, stability and/or function when experimentally tested with mimic mutations [35], suggesting that such PTMs contribute to the decline in muscle function that is seen with aging. Although PTMs likely have a broad impact on muscle homeostasis and disease, they may have an even more pervasive role during muscle wasting considering that this condition is characterized by a decrease in protein synthesis and hence a decline in the levels of newly synthesized (unmodified) proteins [4]. However, most research has focused on the analysis of ubiquitination [4], and relatively little is known about the occurrence and functional roles of other PTMs during muscle wasting. By analysing the regulation of the eight most prevalent/interesting PTMs during muscle wasting induced by cancer, dexamethasone and aging, our study provides key insight into the PTMs that characterize different types of muscle wasting (Figures 1 and 2). Interestingly, although most modified peptides were significantly regulated by only a specific stimulus, there was a small subset of significantly modulated peptides with PTMs that were cross‐shared by 2/3 atrophic stimuli. Remarkably, only 10 of such PTM‐carrying peptides were regulated by all three modes of atrophy, although sometimes in a discordant manner (Figure 3). A decline in Lrpprc dihydroxylation at P27 was one of the four PTMs that were significantly and concordantly regulated by all three atrophic stimuli. The other three PTMs that are consistently modulated during muscle wasting are Atp2a1^G386^ oxidation, Myh7b^T955^ phosphorylation and Myh4^Y1492^ dihydroxylation: these PTMs increase in atrophic vs. control conditions and may contribute to the decline in muscle strength that is observed during cancer cachexia, dexamethasone treatment and aging.

Atp2a1/Serca1 is a sarcoplasmic reticulum (SR) ATPase that pumps Ca^2+^ from the cytosol into the SR lumen, a process essential for muscle relaxation following contraction [36]. The G386 residue is located in the large globular cytoplasmic head of Atp2a1/Serca1, which contains the catalytic site. Previous studies demonstrated that Atp2a1/Serca1 can be inactivated by oxidative modifications, leading to elevated cytosolic Ca^2+^ levels and impaired excitation–contraction coupling [36]. On this basis, increased oxidation of Atp2a1/Serca1^G386^ may contribute to muscle weakness during cancer cachexia, dexamethasone treatment and aging.

Muscle force production may also be impacted by Myh7b^T955^ phosphorylation and Myh4^Y1492^ dihydroxylation because PTM of the rod region of these myosin heavy chain proteins may directly impact their contractile properties, as previously shown for other PTMs [35].

Apart from serving as biomarkers, our experimental analysis of Lrpprc further suggests that some of the wasting‐associated PTMs may causally contribute to muscle weakness (Figures 4, 5, 6, 7, 8).

Lrpprc, also known as Lrp130, is a mRNA binding protein and transcriptional coactivator that is mutated in patients with French–Canadian Leigh syndrome [16, 17, 18]. Previous studies found that Lrpprc is located primarily in the nucleus and mitochondria and that it modulates the expression and stability of mitochondrial mRNAs in multiple tissues (including skeletal muscle), leading to partial defects in the assembly and function of mitochondrial Complex IV (cytochrome c oxidase) [19, 20, 21]. Although Lrpprc normally binds and stabilizes mitochondrial mRNAs, disease‐associated mutations reduce Lrpprc levels, and this impairs the assembly and function of mitochondrial Complex IV and also causes Complex I deficiency [31]. Mechanistically, Lrpprc in complex with Slirp regulates the stability of mitochondrial mRNAs, and disease‐associated mutations impact the polyadenylation of mitochondrial mRNAs in a tissue‐specific manner [19].

Additional functions of Lrpprc were also reported, such as its capacity to suppress autophagy in cell culture [37] and in macrophages of atherosclerotic plaques in mice [38]. In these studies, it was found that Lrpprc interacts with and stabilizes Bcl2, which sequesters Beclin 1. When Lrpprc levels decline, Beclin 1 is released from this complex and can now interact with the class III PI3K Vps34 to form an autophagy‐initiating complex [37].

Apart from its functions in mitochondria and autophagy, Lrpprc also plays important roles in the nucleus, as found from its capacity to interact with PGC‐1α (peroxisome proliferator‐activated receptor gamma coactivator 1α). Specifically, Lrpprc mutation impairs PGC‐1α–mediated metabolic adaptations to fasting in the liver [32]. In particular, Lrpprc is necessary for the transcriptional induction by PGC‐1α of PEPCK (phosphoenolpyruvate carboxykinase), G6P (glucose‐6‐phosphatase) and several mitochondrial genes [32].

Beyond the neurodevelopmental and metabolic symptoms associated with Leigh syndrome, Lrpprc has important physiological functions also in skeletal muscle, where Lrpprc levels are higher in oxidative vs. glycolytic myofibres and positively correlate with PGC‐1α levels. Moreover, it was recently reported that the Lrpprc/Slirp complex regulates mitochondrial structure and respiration [39], and Lrpprc was identified as a regulator of muscle fitness from human GWAS studies [40].

Our analysis of TA muscles that express the dihydroxylation‐resistant mutant Lrpprc^P27A^ indicates that P27 dihydroxylation of Lrpprc sustains muscle force production in young and old age (Figures 4, 8) and that therefore its decline in response to cancer, dexamethasone and aging (Figure 3) may contribute to the reduction in muscle strength that is seen in these conditions. Mechanistically, in parallel with a decline in muscle force, Lrpprc^P27A^ reduces the levels of several mRNAs compared to Lrpprc^WT^ (Figure 6) but appears to be dispensable for mitochondrial function (Supplementary Information Figure S6), autophagy (Supplementary Information Figure S8) and myofibre size in young age (Figure 5) but not in old age (Figure 8). Some of the Lrpprc^P27A^‐down‐regulated mRNAs (such as the *Aplnr* and *Col6*) have widely recognized roles in ensuring muscle function and therefore likely contribute to the decline in muscle strength that is observed in muscles that express Lrpprc^P27A^ vs. Lrpprc^WT^. The decline in the levels of these mRNAs may stem from their decreased expression, consistent with the previously reported role of Lrpprc as a transcriptional co‐regulator of PGC‐1α and potentially of other transcription factors [32]. Alternatively, Lrpprc may bind and stabilize these mRNAs and hence post‐transcriptionally modulate their levels, as previously observed for a subset of mitochondrial mRNAs [19]. Although the vast majority of Lrpprc^P27A^‐modulated genes were down‐regulated, there was also a group of Lrpprc^P27A^–up‐regulated genes (including the E3 ligase *Fbxo32/atrogin‐1*, a widely‐used marker of muscle atrophy [4]), which may also contribute to the decline in muscle force seen in Lrpprc^P27A^ vs. Lrpprc^WT^ muscles. Collectively, these findings suggest that the atrophy‐associated decline in Lrpprc P27 dihydroxylation impairs muscle function by perturbing the levels of key mRNAs. Moreover, Lrpprc P27 dihydroxylation may serve as a general PTM marker of muscle atrophy, given its consistent down‐regulation in response to cancer, dexamethasone and aging.

Altogether, our study provides a compendium of PMTs associated with muscle wasting and defines modifications that are stimulus‐specific and that may serve as potential biomarkers for muscle wasting and for identifying the triggering cause of atrophy.

## Author Contributions

A.S. and F.A.G. did the experimental analyses; S.P. performed the JUMPptm analysis, with help from Y.F.; Y.‐D.W. analysed RNA‐seq data; F.D. supervised the project and wrote the manuscript with help from M.L. The following authors contributed equally to this work: A.S., F.A.G. and S.P.

## Funding

The authors received no specific funding for this work.

## Ethics Statement

The authors certify that they comply with the ethical guidelines for publication in the *Journal of Cachexia, Sarcopenia and Muscle*.

## Conflicts of Interest

The authors declare no conflicts of interest.

## Supporting information


**Dataset: S1.** JUMPptm results for cancer and dexamethasone.

**Dataset: S2.** JUMPptm results for aging.

**Dataset: S3.** PTM consensus sequences.

**Dataset: S4.** RNA‐seq analysis of LrpprcP27A vs. LrpprcWT.

**Dataset: S5.** Source data file and full scans of western blots.

**Figure S1:** Principal component analysis. (A–C) PCA graphs that display that the PTMs recovered with JUMptm cluster in the expected treatment and control groups for aging (A), cancer (B) and dexamethasone (C).
**Figure S2:** PTM consensus sequences are identified from peptides that are significantly modified by cancer, dexamethasone and aging. The consensus motifs for acetylation, carboxylation, deamidation, dihydroxylation, methylation, oxidation, phosphorylation and ubiquitination were identified with iceLogo. The plots show the frequency of the modified amino acid and its surrounding residues (positions −7 to +7). Amino acids are displayed only if they exhibit statistically significant (*p* < 0.05) enrichment (positive values) or depletion (negative values) at a given position.
**Figure S3:** Modulation of Lrpprc mRNA and protein levels in response to muscle wasting induced by distinct triggers. (A) The mRNA levels of Lrpprc (TPM values), as estimated from RNA‐seq, indicate that there are no significant changes in *Lrpprc* expression in response to muscle atrophy induced by aging, dexamethasone and cancer. (B) There is a significant decline in Lrpprc protein levels in response to aging and dexamethasone but not as a result of cancer. In (A and B), the graphs display the mean ± SD with *n* = 3–5 (as indicated). *p* values were calculated with the unpaired two‐tailed *t* test (*ns*, not significant; *p < 0.05 and **p < 0.01).
**Figure S4:** Electroporation of LrpprcWT does not impact muscle force production compared to control GFP electroporation in young mice. Analysis of the force produced by tibialis anterior (TA) muscles that express LrpprcWT (grey) or control GFP (green). (A) Comparison of the TA muscle weight and TA muscle weight normalized by the length of the tibia bone indicates that this is not significantly affected by LrpprcWT (grey) vs. control GFP (green). (B) Likewise, muscle force measurement indicates that, compared to GFP, LrpprcWT does not impact the twitch force and the specific twitch force (normalized by muscle mass) and the corresponding half‐time relaxation and the time to peak. (C) Similar results were also found when analysing the tetanic force, the specific tetanic force and the corresponding half‐time relaxation and the time to peak, which were not affected in LrpprcWT vs. GFP muscles. In (A–C), the graphs display the mean ± SEM with *n* = 9–10. *p* values were calculated with an unpaired two‐tailed *t* test with Welch's correction (all *p* > 0.05, not significant).
**Figure S5:** Analysis of myofibre size in muscles electroporated with plasmids for GFP vs. LrpprcWT in young and old mice. Myofibre size was determined based on laminin immunoreactivity (which delineates the myofibre boundaries) in TA muscles from young (A–C) and old (D–F) mice. The Feret's minimal diameter (A, D), the cross‐sectional area (B, E) and the number of myofibres (C, F) are not significantly different in GFP (green) vs. LrpprcWT (grey). The graphs display the mean ± SEM with *n* = 4–5. *p* values were calculated with an unpaired two‐tailed *t* test (all *p* > 0.05, not significant).
**Figure S6:** Dispensable role of P27 dihydroxylation for mitochondrial function. (A) Analysis of mitochondrial function in cultured myofibres obtained from tibialis anterior skeletal muscles electroporated with LrpprcP27A vs. LrpprcWT. The Seahorse mito stress test indicates that LrpprcP27A (blue) and LrpprcWT (grey) myofibres do not significantly differ in their mitochondrial function, as estimated from the analysis of the basal and maximal respiration. The graphs display the mean ± SEM from *n* = 10 myofibre cultures per group obtained from tibialis anterior muscles sourced from *n* = 10 independent mice. Statistical analyses were done with the unpaired two‐tailed *t* test. (B) Mitochondrial Complex I and Complex IV activity assays from muscle homogenates indicate a similar function of mitochondrial Complex I and Complex IV (normalized by protein content) in LrpprcP27A (blue) vs. LrpprcWT (grey) TA muscles. The graphs display the mean ± SEM from *n* = 4–5 tibialis anterior muscles per group sourced from independent mice. Statistical analysis was done with the unpaired two‐tailed *t* test. (C) Western blots of TA muscles electroporated with LrpprcP27A (blue) vs. LrpprcWT (grey) and probed with a cocktail of antibodies for labile subunits of electron transport chain complexes. These labile subunits are stable only when assembled into complexes, and therefore, they provide a readout for the formation of mitochondrial Complex I, II, III, IV and V. Ponceau staining and α‐tubulin levels are shown as normalisation controls. These analyses indicate no significant changes in the assembly of mitochondrial complexes. The graphs display the mean ± SEM from *n* = 10 tibialis anterior muscles sourced from *n* = 10 independent mice. Statistical analyses were done with the unpaired two‐tailed *t* test. (D) Western blots of TA muscles electroporated with LrpprcP27A (blue) vs. LrpprcWT (grey) indicate no significant changes in the levels of the transcription factor PGC‐1α, a master regulator of mitochondrial function and biogenesis which was previously found to interact with Lrpprc. The graphs display the mean ± SEM from *n* = 10 tibialis anterior muscles sourced from *n* = 10 independent mice. Statistical analyses were done with the paired two‐tailed *t* test.
**Figure S7:** LrpprcWT vs. GFP up‐regulation does impact the assembly status of electron transport chain complexes in the muscles of young mice. Western blots of TA muscles (from young mice) electroporated with control GFP (grey) and LrpprcWT (blue) and probed with a cocktail of antibodies for labile subunits of electron transport chain complexes. This antibody set provides a readout for the formation of mitochondrial Complex I, II, III, IV and V. Ponceau staining and α‐tubulin levels are shown as normalisation controls. These analyses indicate that there are no significant changes in the assembly of mitochondrial complexes when comparing TA muscles electroporated with LrpprcWT vs. control GFP. The graphs display the mean ± SEM from *n* = 5 tibialis anterior muscles sourced from *n* = 5 independent mice. Statistical analysis was done with the unpaired two‐tailed *t* test with Welch's correction (all *p* > 0.05, not significant).
**Figure S8:** Proteostasis markers are not differentially regulated by LrpprcP27A vs. LrpprcWT in the muscles of young mice. Western blot analysis of total homogenates from skeletal muscles electroporated with LrpprcP27A (blue) vs. LrpprcWT (grey). Quantification indicates that there are no changes in the levels of ubiquitinated proteins and in autophagy markers (normalized by total protein levels, i.e., Ponceau staining). Specifically, the conversion of LC3‐I to LC3‐II is not affected, as indicated by similar levels in LC3‐I, LC3‐II and in the LC3‐II/LC3‐I ratio. Moreover, also the levels of phosphorylated Atg16 (pATG16L1), which indicate the rate of autophagy initiation, do not change when comparing LrpprcP27A vs. LrpprcWT muscles from young mice. The graphs display the mean ± SEM from *n* = 10 tibialis anterior muscles sourced from *n* = 10 independent mice. Statistical analysis was done with the paired two‐tailed *t* test (all *p* > 0.05, not significant).
**Figure S9:** Proteostasis markers are not differentially regulated by LrpprcWT vs. GFP in the muscles of young mice. Western blot analysis of total homogenates from skeletal muscles electroporated with LrpprcWT vs. GFP. Quantification indicates that there are no changes in the levels of PGC1‐α, ubiquitinated proteins and autophagy markers (normalized by total protein levels, i.e., Ponceau staining). Specifically, the conversion of LC3‐I to LC3‐II is not affected, as indicated by similar levels in LC3‐I, LC3‐II and in the LC3‐II/LC3‐ I ratio. The graphs display the mean ± SEM from *n* = 5 tibialis anterior muscles sourced from *n* = 5 independent mice. Statistical analysis was done with the unpaired two‐tailed *t* test with Welch's correction (all *p* > 0.05, not significant).
**Figure S10:** Expression of LrpprcP27A‐modulated genes in response to aging, cancer and dexamethasone. Some of the genes that are modulated by LrpprcP27A vs. LrpprcWT are similarly modulated by aging, cancer and/or dexamethasone vs. control conditions, although only some of these genes are significantly regulated. The graphs display the mean ± SEM and *n* = 3. *p* values were calculated with an unpaired two‐tailed *t* test; *p < 0.05, **p < 0.01, ***p < 0.01.
**Figure S11:** Minor effects of LrpprcWT vs. GFP electroporation in the TA muscles of old mice. DNA plasmids that encode for LrpprcWT and GFP were electroporated into the TA muscles of 27‐month‐old male mice. (A) LrpprcWT reduces the TA muscle mass (also when normalized by the length of the tibia bone) compared to GFP, but this has no effect on (B) the twitch muscle force, the specific twitch muscle force, the halftime relaxation and the time to peak. (C) The specific tetanic force increases in LrpprcWT vs. GFP, whereas the tetanic force, the half‐time relaxation and the time to peak are not affected. In (A–C), the graphs display the mean ± SEM with *n* = 4–8 biological replicates (as indicated), and *p* values were calculated with the unpaired two‐tailed Welch's *t* test (*p < 0.05). (D and E) Additional analyses indicate that the specific force (but not the force) increases in the initial phase of the fatigue (D) and force–frequency (E) protocols. The graphs display the mean ± SEM with *n* = 4–7 (D) and *n* = 4–8 (E) biological replicates; two‐way ANOVA was used for the statistical analysis (*p < 0.05).
**Figure S12:** LrpprcP27A does impact the assembly status of electron transport chain complexes in the muscles of old mice. Western blots of TA muscles (from old mice) electroporated with LrpprcP27A (blue) vs. LrpprcWT (grey) and probed with a cocktail of antibodies for labile subunits of electron transport chain complexes; α‐tubulin levels are shown as normalisation controls. There are no significant changes in the assembly of mitochondrial complexes when comparing TA muscles electroporated with LrpprcP27A vs. LrpprcWT. The graphs display the mean ± SEM from *n* = 4 tibialis anterior muscles. Statistical analysis was done with the paired two‐tailed *t* test (all *p* > 0.05, not significant).
**Figure S13:** Proteostasis markers are not differentially regulated by LrpprcP27A vs. LrpprcWT in the muscles of old mice. Western blot analysis of total homogenates from skeletal muscles electroporated with LrpprcP27A (blue) vs. LrpprcWT (grey). Quantification indicates that there are no changes in the levels of PGC1‐α, ubiquitinated proteins and autophagy markers (normalized by tubulin levels). Specifically, the levels of phosphorylated Atg16 (pATG16L1) and the conversion of LC3‐I to LC3‐II are not affected, as indicated by similar levels in LC3‐I, LC3‐II and in the LC3‐II/LC3‐I ratio. The graphs display the mean ± SEM from *n* = 4 tibialis anterior muscles. Statistical analysis was done with the paired two‐tailed *t* test (all *p* > 0.05, not significant).

## Data Availability

The JUMPptm results are reported in Datasets S1 and S2. The PTM consensus sequences are reported in Dataset S3, and the RNA‐seq data are provided in Dataset S4. Additional data and the full scans of western blots are provided in the Source Data file, Dataset S5. The RNA‐seq data of gene expression changes induced by Lrpprc^P27A^ vs. Lrpprc^WT^ have been deposited to the Gene Expression Omnibus (GEO) and are accessible with the identifier GSE296017. The RNA‐seq data from mouse muscles with different types of atrophy (aging, cancer and dexamethasone) were previously published [12] and are accessible at the GEO with the identifier GSE159952.
