# Supplementary material for: Largely Distinct Post‐Translational Modifications Differentiate Skeletal Muscle Wasting Caused by Cancer, Dexamethasone and Aging
Source: J Cachexia Sarcopenia Muscle. 2026 Feb 4;17(1):e70220. doi: 10.1002/jcsm.70220 (PMC12872332; doi:10.1002/jcsm.70220)
Supplement: Supplementary file 6 — Figure S1: Principal component analysis. (A–C) PCA graphs that display that the PTMs recovered with JUMptm cluster in the expected treatment and control groups for aging (A), cancer (B) and dexamethasone (C). Figure S2: PTM consensus sequences are identified from peptides that are significantly modified by cancer, dexamethasone and aging. The consensus motifs for acetylation, carboxylation, deamidation, dihydroxylation, methylation, oxidation, phosphorylation and ubiquitination were identified with iceLogo. The plots show the frequency of the modified amino acid and its surrounding residues (positions −7 to +7). Amino acids are displayed only if they exhibit statistically significant (p < 0.05) enrichment (positive values) or depletion (negative values) at a given position. Figure S3: Modulation of Lrpprc mRNA and protein levels in response to muscle wasting induced by distinct triggers. (A) The mRNA levels of Lrpprc (TPM values), as estimated from RNA‐seq, indicate that there are no significant changes in Lrpprc expression in response to muscle atrophy induced by aging, dexamethasone and cancer. (B) There is a significant decline in Lrpprc protein levels in response to aging and dexamethasone but not as a result of cancer. In (A and B), the graphs display the mean ± SD with n = 3–5 (as indicated). p values were calculated with the unpaired two‐tailed t test (ns, not significant; *p < 0.05 and **p < 0.01). Figure S4: Electroporation of LrpprcWT does not impact muscle force production compared to control GFP electroporation in young mice. Analysis of the force produced by tibialis anterior (TA) muscles that express LrpprcWT (grey) or control GFP (green). (A) Comparison of the TA muscle weight and TA muscle weight normalized by the length of the tibia bone indicates that this is not significantly affected by LrpprcWT (grey) vs. control GFP (green). (B) Likewise, muscle force measurement indicates that, compared to GFP, LrpprcWT does not impact the twitch force and the [file JCSM-17-e70220-s002.pdf]

SUPPORTING INFORMATION

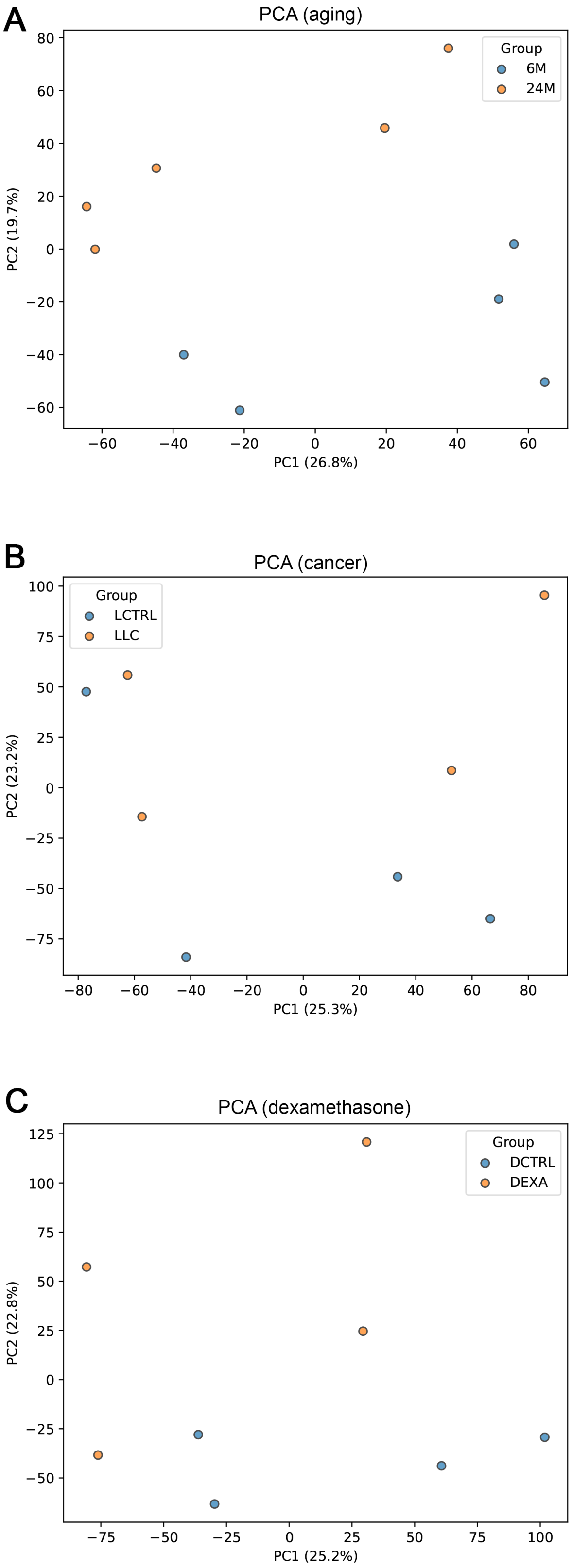

Supplementary Figure S1 | Principal Component Analysis. (**A-C**) PCA graphs that display that the PTMs recovered with JUMptm cluster in the expected treatment and control groups for aging (A), cancer (B), and dexamethasone (C).

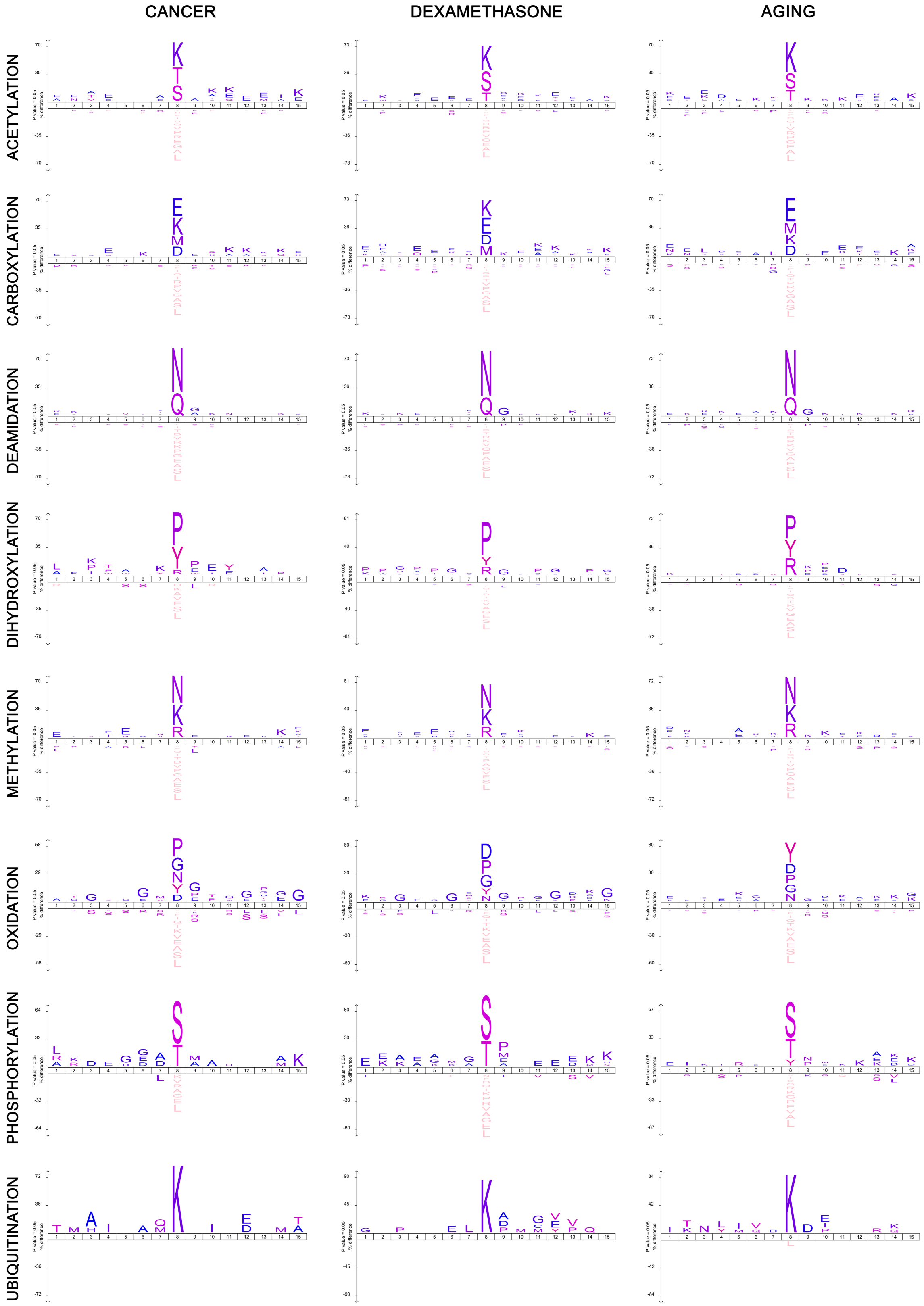

Supplementary Figure S2 | PTM consensus sequences are identified from peptides that are significantly modified by cancer, dexamethasone, and aging. The consensus motifs for acetylation, carboxylation, deamidation, dihydroxylation, methylation, oxidation, phosphorylation, and ubiquitination were identified with iceLogo. The plots show the frequency of the modified amino acid and its surrounding residues (positions −7 to +7). Amino acids are displayed only if they exhibit statistically significant (P<0.05) enrichment (positive values) or depletion (negative values) at a given position.

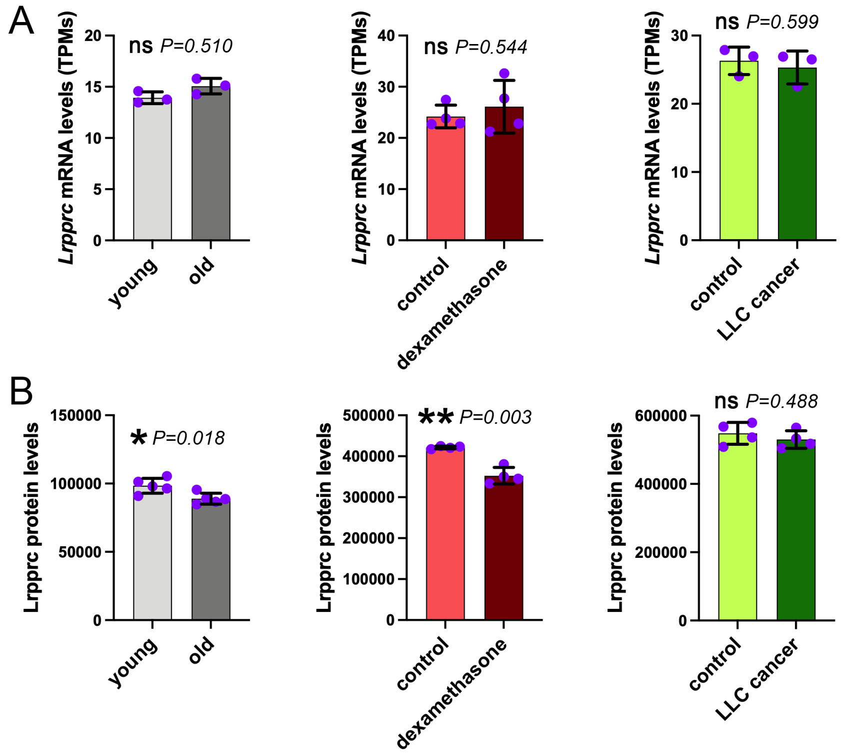

Supplementary Figure S3 | Modulation of Lrpprc mRNA and protein levels in response to muscle wasting induced by distinct triggers. (A) The mRNA levels of Lrpprc (TPM values), as estimated from RNA-seq, indicate that there are no significant changes in *Lrpprc* expression in response to muscle atrophy induced by aging, dexamethasone, and cancer. (B) There is a significant decline in Lrpprc protein levels in response to aging and dexamethasone but not as a result of cancer. In (A-B), the graphs display the mean ± SD with n=3-5 (as indicated). *P*-values were calculated with the unpaired two-tailed t-test (*ns*, not significant; **P*<0.05, and ***P*<0.01).

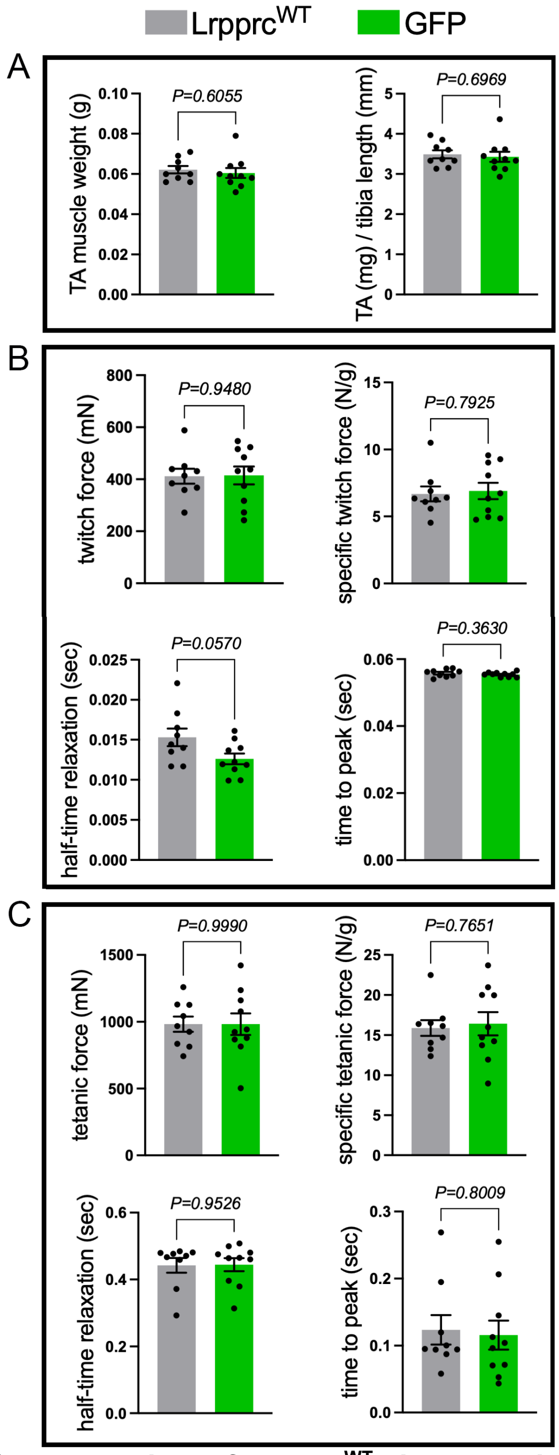

Supplementary Figure S4 | Electroporation of Lrpprc^{WT} does not impact muscle force production compared to control GFP electroporation in young mice. Analysis of the force produced by tibialis anterior (TA) muscles that express Lrpprc^{WT} (gray) or control GFP (green). (A) Comparison of the TA muscle weight and TA muscle weight normalized by the length of the tibia bone indicates that this is not significantly affected by Lrpprc^{WT} (gray) vs. control GFP (green). (B) Likewise, muscle force measurement indicates that, compared to GFP, Lrpprc^{WT} does not impact the twitch force and the specific twitch force (normalized by muscle mass) and the corresponding half-time relaxation and the time to peak. (C) Similar results were also found when analyzing the tetanic force, the specific tetanic force, and the corresponding half-time relaxation and the time to peak, which were not affected in Lrpprc^{WT} vs. GFP muscles. In (A-C), the graphs display the mean ± SEM with n=9-10. *P*-values were calculated with an unpaired two-tailed t-test with Welch's correction (all $P>0.05$, not significant).

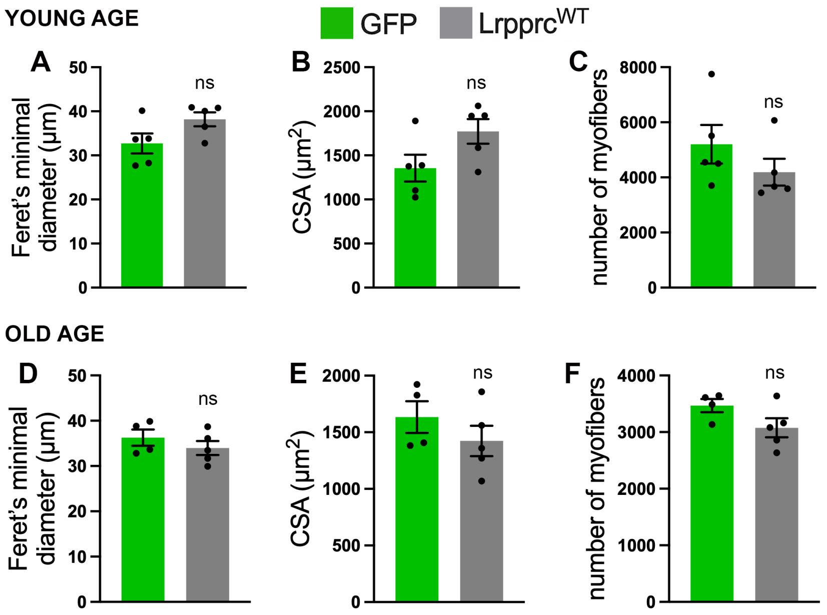

Supplementary Figure S5 | Analysis of myofiber size in muscles electroporated with plasmids for GFP vs. Lrpprc^{WT} in young and old mice. Myofiber size was determined based on laminin immunoreactivity (which delineates the myofiber boundaries) in TA muscles from young (A-C) and old (D-F) mice. The Feret's minimal diameter (A, D), the cross-sectional area (B, E), and the number of myofibers (C, F) are not significantly different in GFP (green) vs. Lrpprc^{WT} (gray). The graphs display the mean ± SEM with n=4-5. *P*-values were calculated with an unpaired two-tailed t-test (all $P>0.05$, not significant).

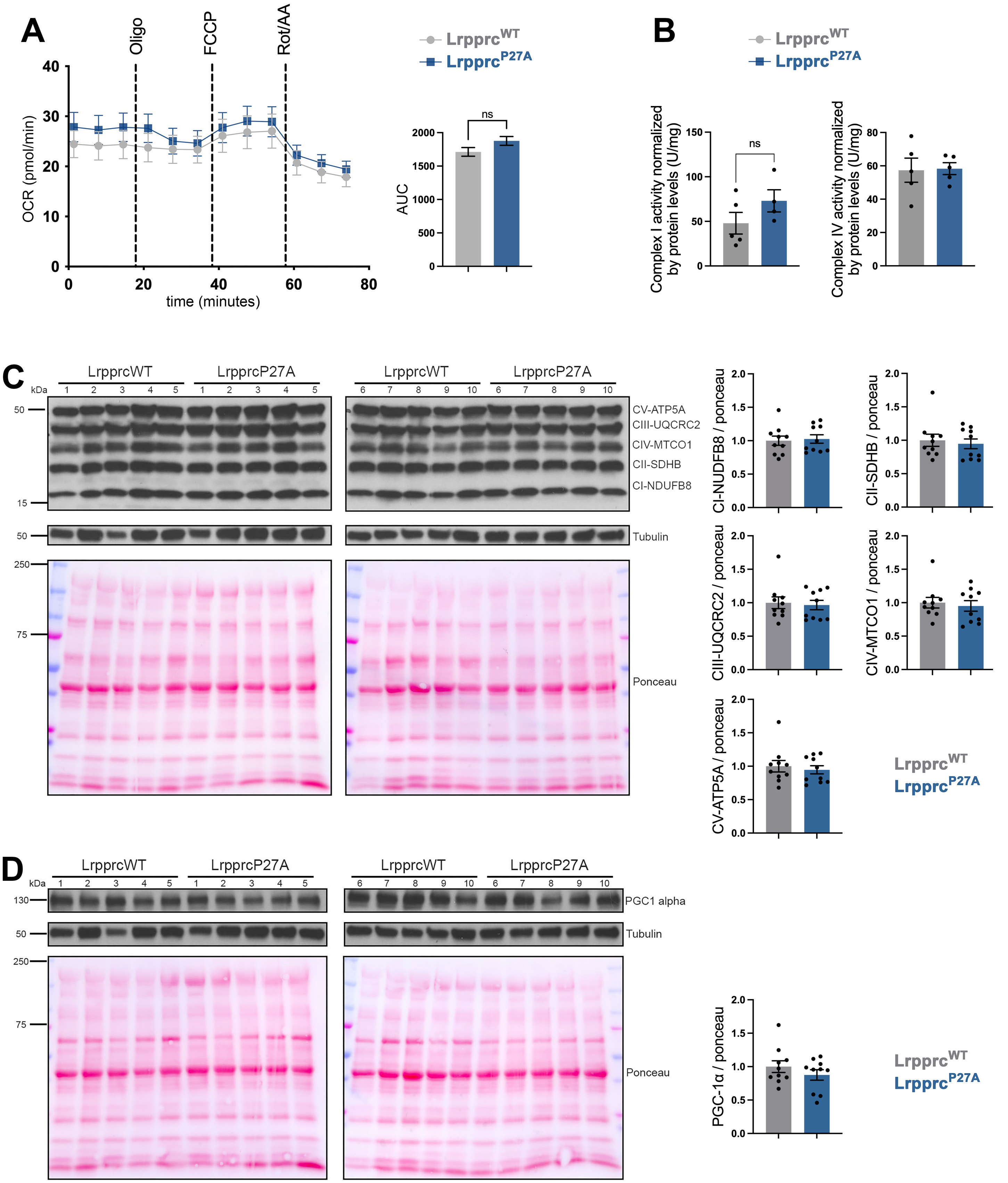

Supplementary Figure S6 | Dispensable role of P27 dihydroxylation for mitochondrial function. (A) Analysis of mitochondrial function in cultured myofibers obtained from tibialis anterior skeletal muscles electroporated with Lrpprc^{P27A} vs. Lrpprc^{WT}. The Seahorse mito stress test indicates that Lrpprc^{P27A} (blue) and

Lrpprc^{WT} (gray) myofibers do not significantly differ in their mitochondrial function, as estimated from the analysis of the basal and maximal respiration. The graphs display the mean ±SEM from n=10 myofiber cultures per group obtained from tibialis anterior muscles sourced from n=10 independent mice. Statistical analyses were done with the unpaired two-tailed t-test. (B) Mitochondrial complex I and complex IV activity assays from muscle homogenates indicate a similar function of mitochondrial complex I and IV (normalized by protein content) in Lrpprc^{P27A} (blue) vs. Lrpprc^{WT} TA muscles. The graphs display the mean ±SEM from n=4-5 tibialis anterior muscles per group sourced from independent mice. Statistical analysis was done with the unpaired two-tailed t-test.

(C) Western blots of TA muscles electroporated with Lrpprc^{P27A} (blue) vs. Lrpprc^{WT} (gray) and probed with a cocktail of antibodies for labile subunits of electron transport chain complexes. These labile subunits are stable only when assembled into complexes and therefore they provide a readout for the formation of mitochondrial complex I, II, III, IV, and V. Ponceau staining and α-tubulin levels are shown as normalization controls. These analyses indicate no significant changes in the assembly of mitochondrial complexes. The graphs display the mean ±SEM from n=10 tibialis anterior muscles sourced from n=10 independent mice. Statistical analyses were done with the unpaired two-tailed t-test.

(D) Western blots of TA muscles electroporated with Lrpprc^{P27A} (blue) vs. Lrpprc^{WT} (gray) indicate no significant changes in the levels of the transcription factor PGC-1α, a master regulator of mitochondrial function and biogenesis which was previously found to interact with Lrpprc. The graphs display the mean ±SEM from n=10 tibialis anterior muscles sourced from n=10 independent mice. Statistical analyses were done with the paired two-tailed t-test.

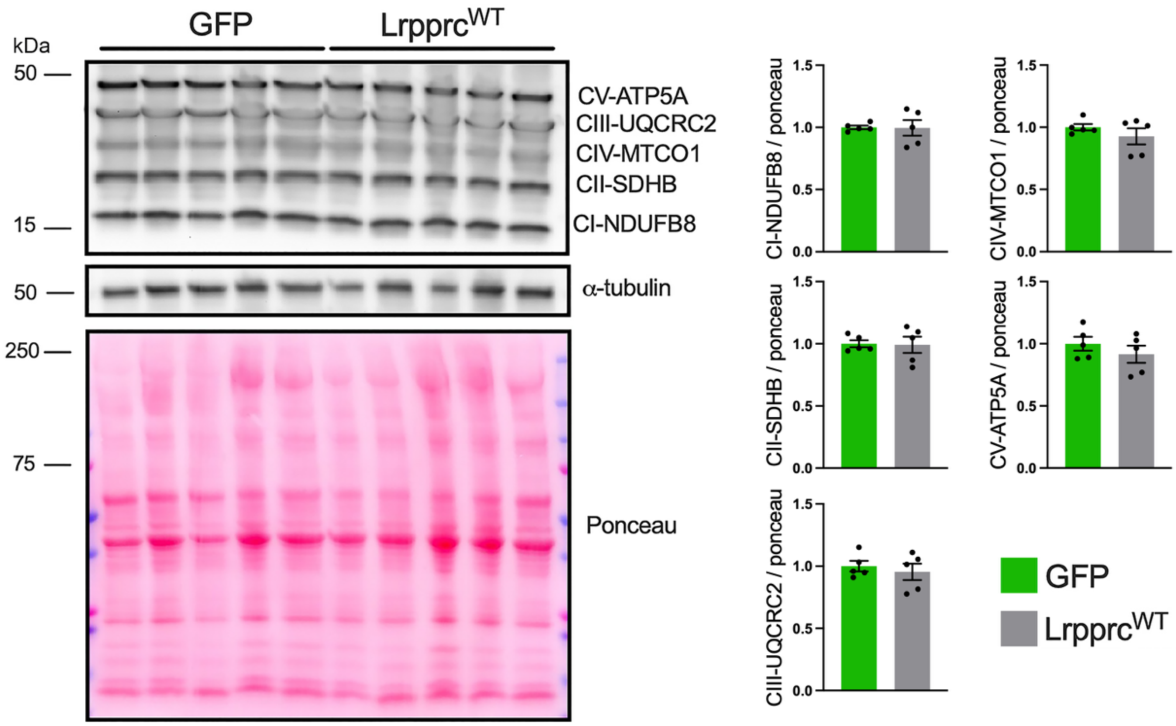

Supplementary Figure S7 | Lrpprc^{WT} vs. GFP upregulation does impact the assembly status of electron transport chain complexes in the muscles of young mice. Western blots of TA muscles (from young mice) electroporated with control GFP (gray) and Lrpprc^{WT} (blue) and probed with a cocktail of antibodies for labile subunits of electron transport chain complexes. This antibody set provides a readout for the formation of mitochondrial complex I, II, III, IV, and V. Ponceau staining and α-tubulin levels are shown as normalization controls. These analyses indicate that there are no significant changes in the assembly of mitochondrial complexes when comparing TA muscles electroporated with Lrpprc^{WT} vs. control GFP. The graphs display the mean ±SEM from n=5 tibialis anterior muscles sourced from n=5 independent mice. Statistical analysis was done with the unpaired two-tailed t-test with Welch's correction (all P>0.05, not significant).

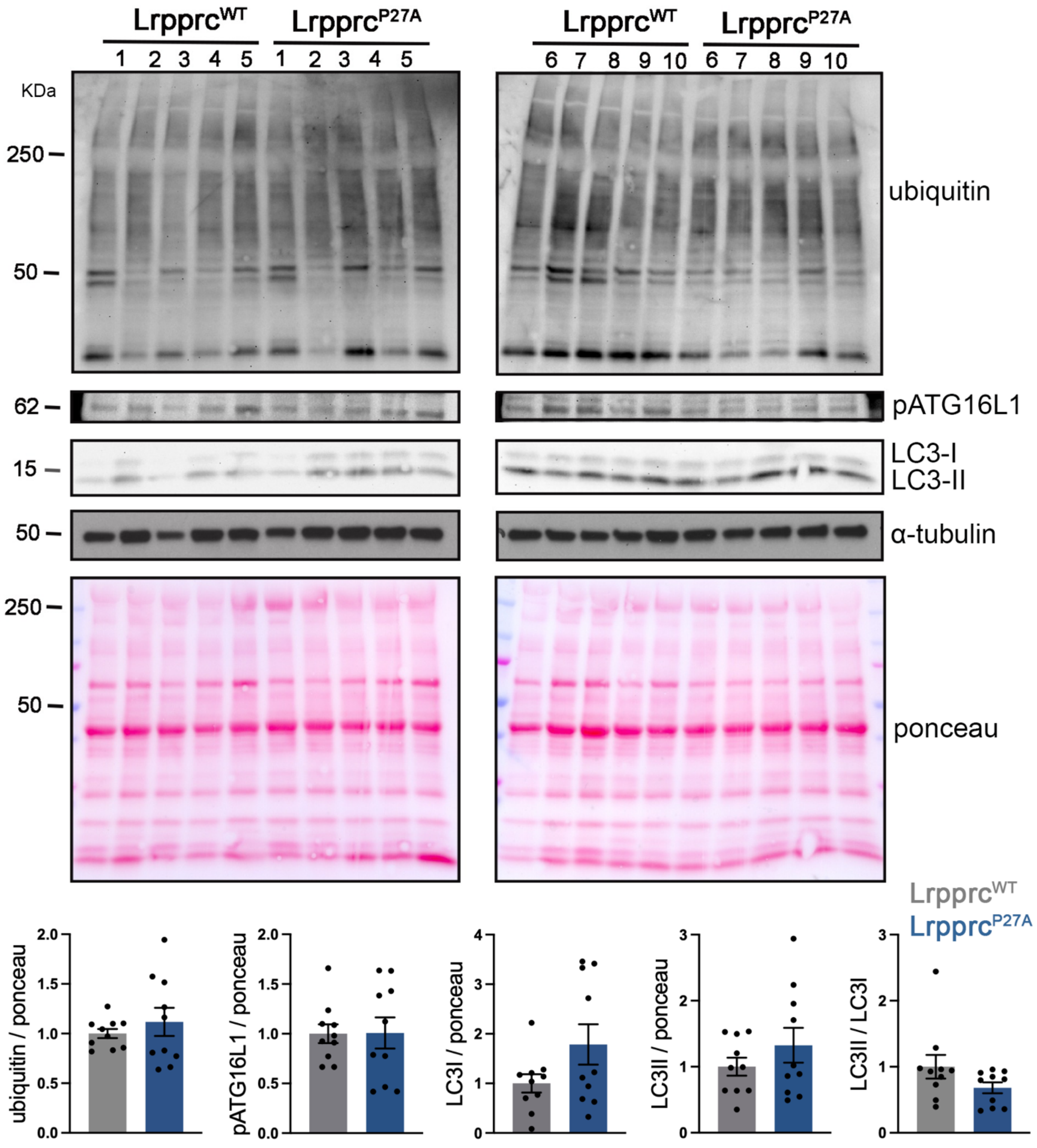

Supplementary Figure S8 | Proteostasis markers are not differentially regulated by Lrpprc^{P27A} vs. Lrpprc^{WT} in the muscles of young mice. Western blot analysis of total homogenates from skeletal muscles electroporated with Lrpprc^{P27A} (blue) vs. Lrpprc^{WT} (gray). Quantification indicates that there are no changes in the levels of ubiquitinated proteins and in autophagy markers (normalized by total protein levels, i.e. ponceau staining). Specifically, the conversion of LC3-I to LC3-II is not affected, as indicated by similar levels in LC3-I, LC3-II, and in the LC3-II/LC3-I ratio. Moreover, also the levels of phosphorylated Atg16 (pATG16L1), which indicate the rate of autophagy initiation, do not change when comparing Lrpprc^{P27A} vs. Lrpprc^{WT} muscles from young mice. The graphs display the mean ±SEM from n=10 tibialis anterior muscles sourced from n=10 independent mice. Statistical analysis was done with the paired two-tailed t-test (all *P*>0.05, not significant).

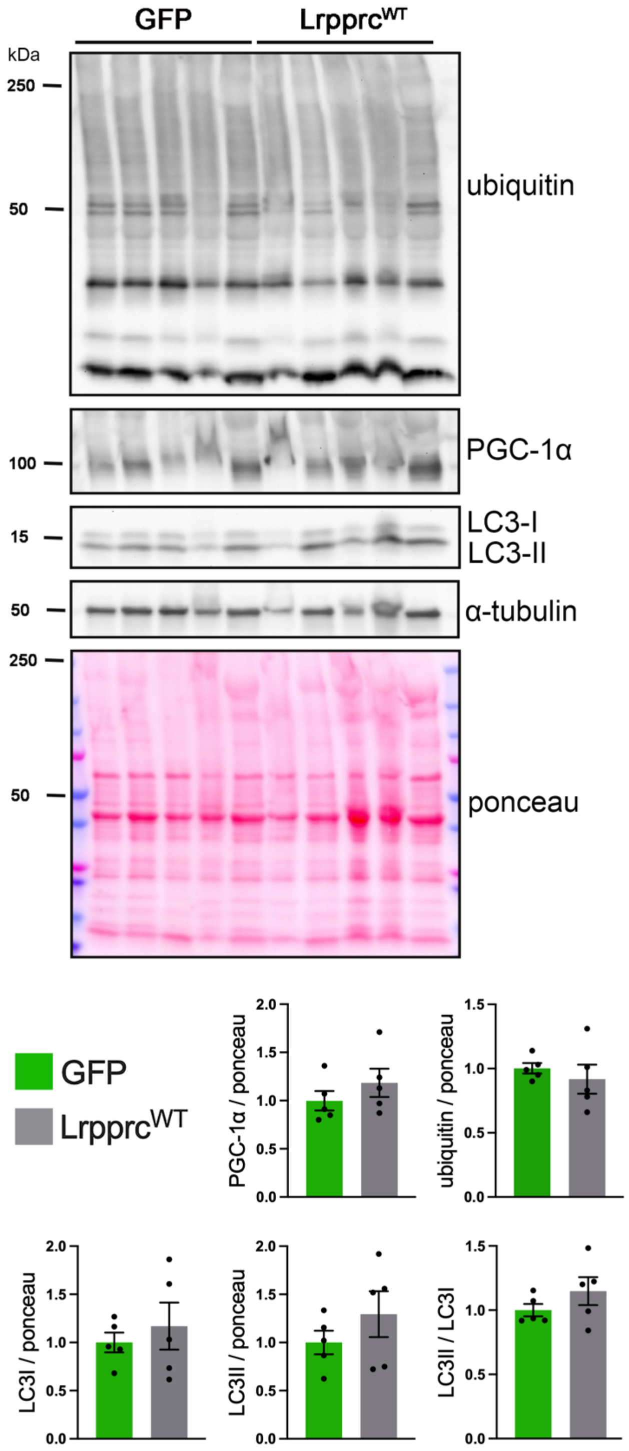

Supplementary Figure S9 | Proteostasis markers are not differentially regulated by Lrpprc^{WT} vs. GFP in the muscles of young mice. Western blot analysis of total homogenates from skeletal muscles electroporated with Lrpprc^{WT} vs. GFP. Quantification indicates that there are no changes in the levels of PGC1-α, ubiquitinated proteins, and autophagy markers (normalized by total protein levels, i.e. ponceau staining). Specifically, the conversion of LC3-I to LC3-II is not affected, as indicated by similar levels in LC3-I, LC3-II, and in the LC3-II/LC3-I ratio. The graphs display the mean ±SEM from n=5 tibialis anterior muscles sourced from n=5 independent mice. Statistical analysis was done with the unpaired two-tailed t-test with Welch's correction (all $P>0.05$, not significant).

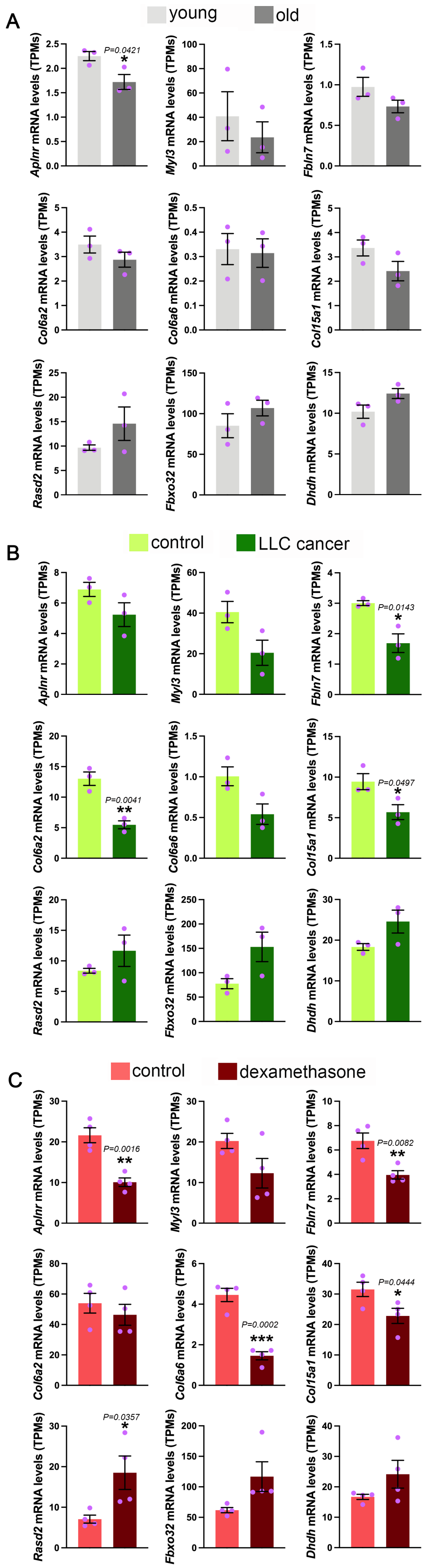

Supplementary Figure S10 | Expression of Lrpprc^{P27A}-modulated genes in response to aging, cancer, and dexamethasone. Some of the genes that are modulated by Lrpprc^{P27A} vs. Lrpprc^{WT} are similarly modulated by aging, cancer, and/or dexamethasone vs. control conditions, although only some of these genes are significantly regulated. The graphs display the mean ± SEM and n=3. *P*-values were calculated with an unpaired two-tailed t-test; **P*<0.05, ***P*<0.01, ****P*<0.01.

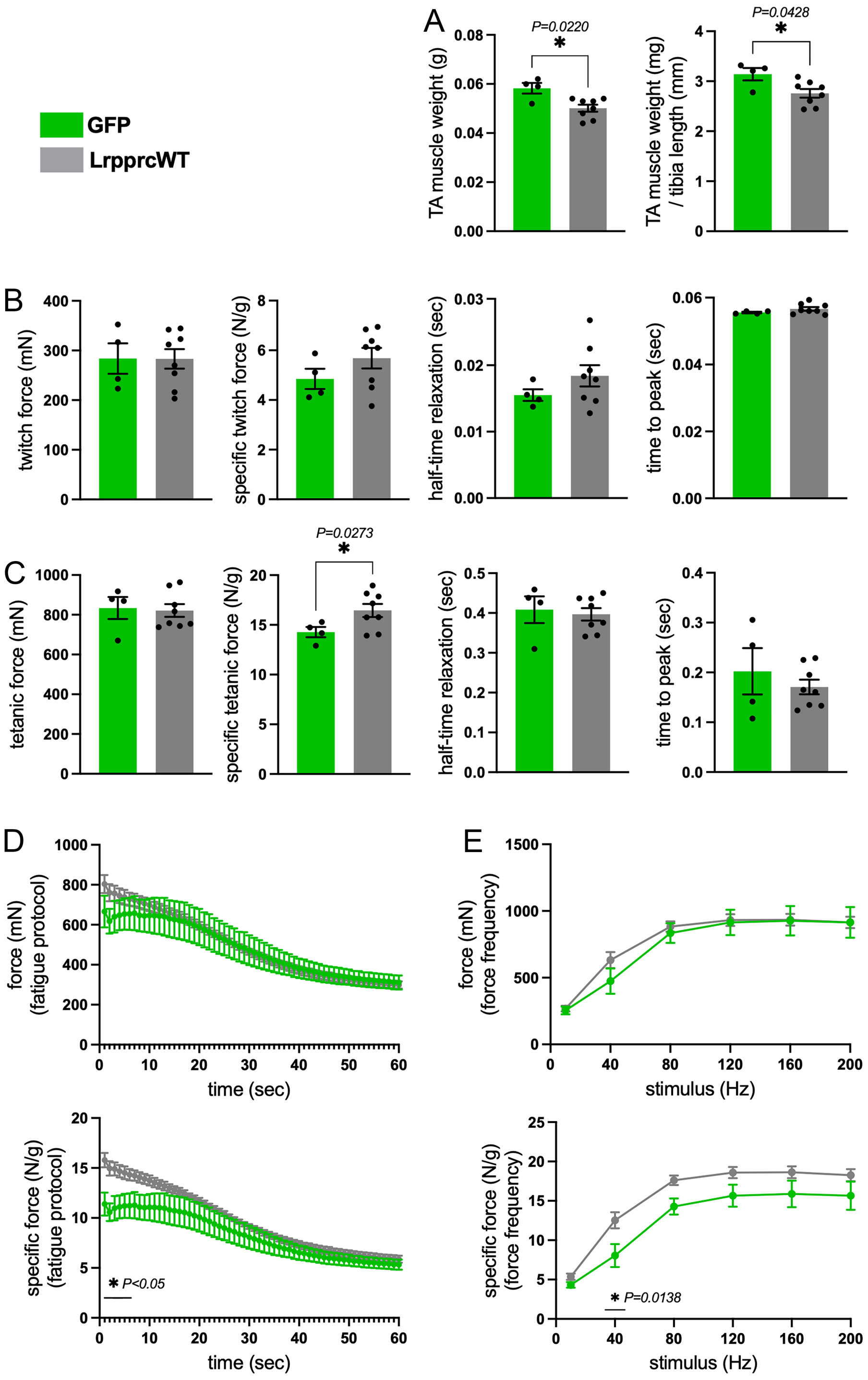

Supplementary Figure S11 | Minor effects of Lrpprc^{WT} vs. GFP electroporation in the TA muscles of old mice. DNA plasmids that encode for Lrpprc^{WT} and GFP were electroporated into the TA muscles of 27-month-old male mice. (A) Lrpprc^{WT} reduces the TA muscle mass (also when normalized by the length of the tibia bone) compared to GFP but this has no effect on (B) the twitch muscle force, the specific twitch muscle force, the half-time relaxation, and the time to peak. (C) The specific tetanic force increases in Lrpprc^{WT} vs. GFP whereas the tetanic force, the half-time relaxation, and the time to peak are not affected. In (A-C), the graphs display the mean ± SEM with n=4-8 biological replicates (as indicated), and *P*-values were calculated with the unpaired two-tailed Welch's t-test (**P*<0.05). (D-E) Additional analyses indicate that the specific force (but not the force) increases in the initial phase of the fatigue (D) and force frequency (E) protocols. The graphs display the mean ± SEM with n=4-7 (D) and n=4-8 (E) biological replicates; two-way ANOVA was used for the statistical analysis (**P*<0.05).

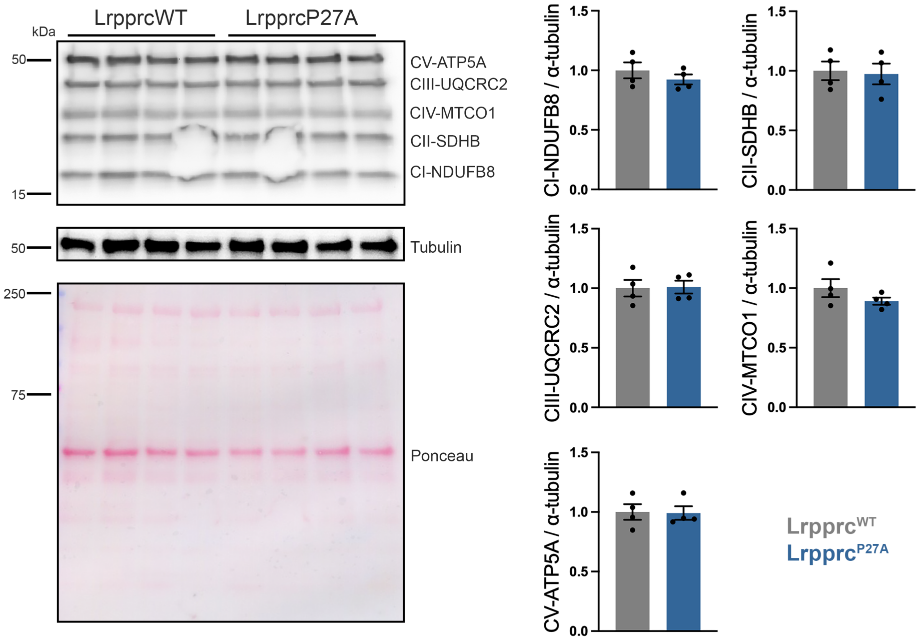

Supplementary Figure S12 | Lrpprc^{P27A} does impact the assembly status of electron transport chain complexes in the muscles of old mice. Western blots of TA muscles (from old mice) electroporated with Lrpprc^{P27A} (blue) vs. Lrpprc^{WT} (gray) and probed with a cocktail of antibodies for labile subunits of electron transport chain complexes; α-tubulin levels are shown as normalization controls. There are no significant changes in the assembly of mitochondrial complexes when comparing TA muscles electroporated with Lrpprc^{P27A} vs. Lrpprc^{WT}. The graphs display the mean ±SEM from n=4 tibialis anterior muscles. Statistical analysis was done with the paired two-tailed t-test (all P>0.05, not significant).

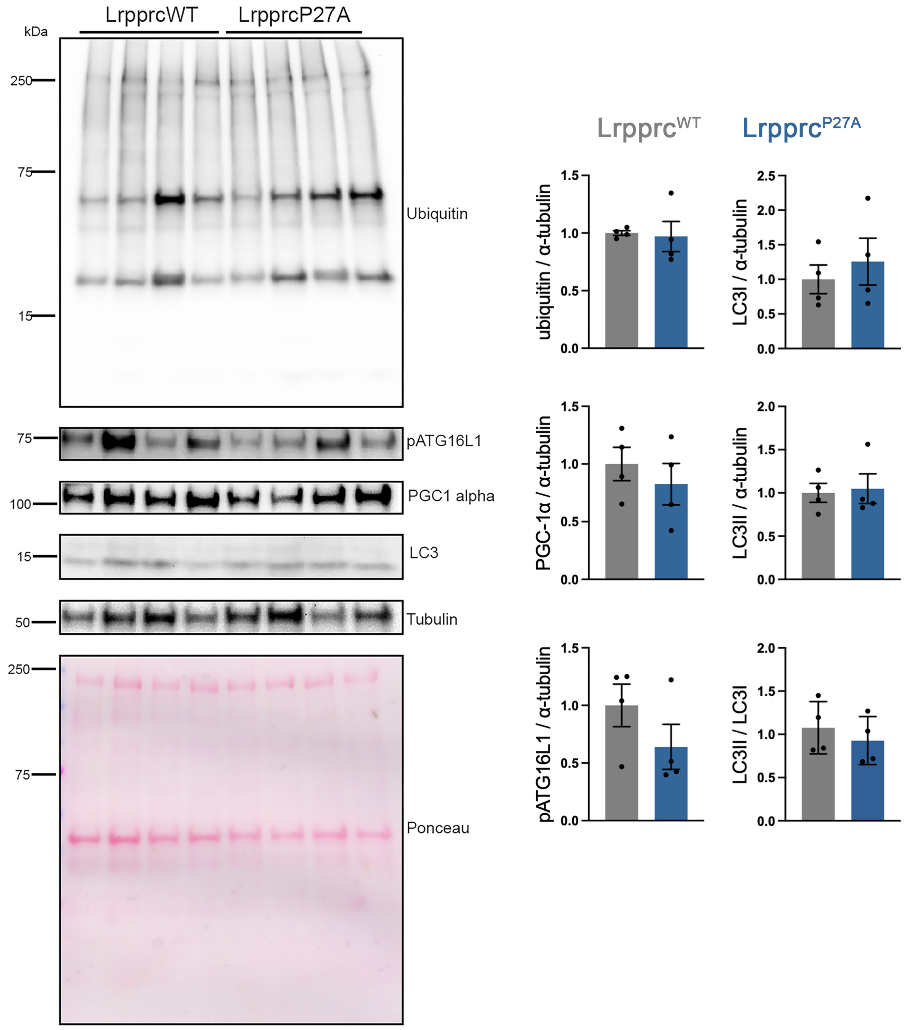

Supplementary Figure S13 | Proteostasis markers are not differentially regulated by Lrpprc^{P27A} vs. Lrpprc^{WT} in the muscles of old mice. Western blot analysis of total homogenates from skeletal muscles electroporated with Lrpprc^{P27A} (blue) vs. Lrpprc^{WT} (gray). Quantification indicates that there are no changes in the levels of PGC1-α, ubiquitinated proteins, and autophagy markers (normalized by tubulin levels). Specifically, the levels of phosphorylated Atg16 (pATG16L1) and the conversion of LC3-I to LC3-II is not affected, as indicated by similar levels in LC3-I, LC3-II, and in the LC3-II/LC3-I ratio. The graphs display the mean ±SEM from n=4 tibialis anterior muscles. Statistical analysis was done with the paired two-tailed t-test (all $P>0.05$, not significant).
